# Induction of meiosis by embryonic gonadal somatic cells differentiated from pluripotent stem cells

**DOI:** 10.1186/s13287-021-02672-4

**Published:** 2021-12-20

**Authors:** Haiying Wang, Linlin Liu, Chang Liu, Lingling Wang, Jiyu Chen, Huasong Wang, Dai Heng, Ming Zeng, Chun Liu, Zhongcheng Zhou, Xiaoying Ye, Yajuan Wan, Huiyu Li, Lin Liu

**Affiliations:** 1 State Key Laboratory of Medicinal Chemical Biology, Nankai University, Tianjin, 300071 China; 2 Department of Cell Biology and Genetics, College of Life Sciences; The Key Laboratory of Bioactive Materials Ministry of Education, Nankai University, Tianjin, 300071 China; 3 The Key Laboratory of Bioactive Materials Ministry of Education, College of Life Sciences, Nankai University, 94 Weijin Road, Tianjin, 300071 China; 4 Department of Cell Biology, College of Life Sciences, Tsinghua University, Beijing, 100084 China; 5 Department of Obstetrics and Gynecology, Center for Reproductive Medicine, Key Laboratory for Major Obstetric Diseases of Guangdong Province, The Third Affiliated Hospital of Guangzhou Medical University, Guangzhou, China; 6 Guangzhou Women and Children’s Medical Center, Guangzhou Medical University, Guangzhou, 510655 China

**Keywords:** ESCs, GSCLCs, Meiosis, Small molecules, Female infertility

## Abstract

**Background:**

Depletion of oocytes leads to ovarian aging-associated infertility, endocrine disruption and related diseases. Excitingly, unlimited oocytes can be generated by differentiation of primordial germ cell like cells (PGCLCs) from pluripotent stem cells. Nevertheless, development of oocytes and follicles from PGCLCs relies on developmentally matched gonadal somatic cells, only available from E12.5 embryos in mice. It is therefore imperative to achieve an in vitro source of E12.5 gonadal somatic cells.

**Methods:**

We explored to identify small molecules, which can induce female embryonic stem cells (ESCs) into gonadal somatic cell like cells.

**Results:**

Using RNA-sequencing, we identified signaling pathways highly upregulated in E12.5_gonadal somatic cells (E12.5_GSCs). Through searching for the activators of these pathways, we identified small-molecule compounds Vitamin C (Vc) and AM580 in combination (V580) for inducing differentiation of female embryonic stem cells (ESCs) into E12.5_GSC-like cells (E12.5_GSCLCs). After V580 treatment for 6 days and sorted by a surface marker CD63, the cell population yielded a transcriptome profile similar to that of E12.5_GSCs, which promoted meiosis progression and folliculogenesis of primordial germ cells. This approach will contribute to the study of germ cell and follicle development and oocyte production and have implications in potentially treating female infertility.

**Conclusion:**

ESCs can be induced into embryonic gonadal somatic cell like cells by small molecules.

**Supplementary Information:**

The online version contains supplementary material available at 10.1186/s13287-021-02672-4.

## Introduction

With the increasing pressure of social competition, many women choose to postpone their childbearing age. Coupled with the influence of diet and environmental factors, the phenomenon of infertility caused by ovarian aging has increased significantly [1–3]. In addition, ovarian aging can lead to early menopause and related chronic diseases, such as coronary heart disease, osteoporosis, and endocrine disorders, which seriously affect female reproduction and physical and mental health [4–7]. Depletion of oocyte and follicle reserve in vivo makes direct contribution to ovarian aging. Hence, extensive efforts have been made over the last decades to generate oocytes in vitro, from other source, such as from pluripotent stem cells.

Close interactions between germ cells and somatic cells are essential for ovarian development and function [8–10], and can control germ cell proliferation, meiotic entry and arrest as well as formation of the primordial follicle pool [11, 12]. Embryonic primordial germ cells (PGCs) undergo migration and proliferation, followed by meiosis, which is arrested at diakinesis during prophase I prior to birth [13–17]. Meiosis continues after puberty, and oocytes develop with granulosa cells during folliculogenesis, giving rise to mature oocytes for reproduction. As the number of germ cells is set at birth in most mammalian species [18–21], current evidence does not support neo-folliculogenesis after the ovarian reserve is determined [22, 23]. Meanwhile, depletion of a limited follicle reserve, together with some uncontrollable factors, such as age, food, and the haze environment, lead to a series of diseases, including endocrine disorders and infertility [1, 2]. Remarkably, oocyte-like cells [24] and PGC-like cells (PGCLCs) [25–29] have been consistently obtained from pluripotent stem cells. Furthermore, PGCLCs and E12.5 PGCs require reconstitution with E12.5_GSCs to enter meiosis and folliculogenesis for production of functional oocytes and, consequently, offspring [27, 29]. This approach holds great promise for the treatment of infertility as well as for restoration of ovarian endocrine function as an alternative to feasible, yet risky methods such as hormone replacement therapy [30–32]. Furthermore, reconstitution of the entire process of gametogenesis has been achieved in vitro, thus, providing a platform for analyzing the mechanistic details of oogenesis [33].

To fulfill the potential of PGCLCs in vitro, their necessary interaction with developmentally matched gonadal somatic cells must be ensured for normal folliculogenesis [34–37]. However, successful PGCs development and maturation experiments currently require embryo destruction to obtain matched somatic cells. Meanwhile, it has been suggested that only E12.5_GSCs can support maturation of nascent PGCs or PGCLCs into mature oocytes [27, 29, 38, 39]. They aggregated PGCs or PGCLCs with E12.5_GSCs to form reconstituted ovaries and transplanted them into ovarian bursa or kidney capsules [38–40]. PGCs in the reconstituted ovaries matured into germinal vesicle-stage oocytes, which then contributed to fertility following in vitro maturation and fertilization [29, 33]. However, to construct a successful platform for elucidating the molecular mechanisms underlying meiosis and oocyte production, developmentally matched gonadal somatic cells are indispensable. These cells have only been obtained from E12.5 female gonads [33], until a recent breakthrough [41]. Moreover, the embryo destruction required for obtaining these cells is not feasible in humans. Therefore, an alternative approach for the generation of these cells in vitro is imperative [33]. Compared with genetic manipulation and difficult-to-manufacture biologics, small molecules offer advantages, including cell permeability, cost-effectiveness, no immunogenicity, simpler synthesis, batch-to-batch consistency, and preservation [42]. In addition, their regulatory effects on protein function are reversible and can be fine-tuned by varying their concentrations [43]. Hence, we sought to identify small-molecule compounds that can stimulate the differentiation of ESCs into E12.5_GSCLCs. Our approach may facilitate further in-depth study of oocyte production.

## Methods

### Mouse embryonic fibroblasts (MEF) isolation and cell culture

All the animal experiments were performed following the ethical guidelines approved by Tianjin Animal Management Committee. MEF cells were derived from E13.5 embryos isolated from B6C3F1 mice via cesarean section and washed in phosphate-buffered saline (PBS). Heads and visceral tissues were removed, and the remaining tissue was washed in PBS, submerged in 0.25% trypsin–EDTA (0.25% TE, Invitrogen), and incubated at 37 °C for 10 min. The tissue was pipetted repeatedly to aid dissociation, washed, and plated in MEF medium, Dulbecco’s modified eagle’s medium (DMEM, Invitrogen) supplemented with 10% fetal bovine serum (FBS, Hyclone), 1 mM L-glutamine (Invitrogen), 1% nonessential amino acid stock (NEAA, Sigma), penicillin (100 U/mL), and streptomycin (100 μg/mL). Cells were cultured at 37 °C in 5% CO_2_ with humidified air (Thermo Scientific, USA). ESC lines were established and characterized based on a previously described method [44], cultured in KnockOut DMEM supplemented with 20% FBS (ES quality, Hyclone), 1000 U/mL leukemia inhibitory factor (LIF) (ESGRO, Chemicon), 0.1 mM nonessential amino acids, 0.1 mM β-mercaptoethanol, 1 mM L-glutamine, penicillin (100 U/mL), and streptomycin (100 μg/mL).

### Magnetic-activating cell sorting (MACS)

MACS was performed according to the manufacturer’s instructions (Miltenyi). Briefly, dissociated E12.5 gonadal cells were treated with 0.05% trypsin–EDTA to harvest single cells, resuspended in 80 μL of MACS buffer containing 0.5% BSA and 2 mM EDTA (pH = 8.0), then incubated with 20 μL anti-SSEA1 antibodies conjugated to magnetic beads (Miltenyi) on ice for 20 min. Cell suspensions were washed in PBS and applied to an MS column to separate SSEA1-positive cells (PGCs) and SSEA1-negative cells (E12.5_GSCs). GSCs were collected from the flow-through. The cells that remained on the column were PGCs. To ensure that PGCs do not contain E12.5_GSCs, we filtered them through the MS column thrice.

### RNA extraction and quantitative real-time PCR (qPCR)

RNA was extracted from samples using RNeasy Mini Kit (Qiagen) according to manufacturer’s method. 2 μg RNA were reversely transcribed into cDNA using M-MLV reverse transcriptase (Invitrogen). Quantitative real-time PCR reactions were set up in duplicate with the Faststart Universal SYBR Green Master Mix (Roche) and run on the real-time PCR Detection System (Bio-Rad). *Gapdh* was served as the internal control. The primers used are listed in Additional file 1: Table S1.

### Induction of GSCLCs from ESCs

Before cell induction, we prepared a 12-well culture plate coated with human plasma fibronectin (HFN, 16.7 mg/mL, Millipore) kept for at least 1 h in a 37 °C CO_2_ incubator. ESCs cultured in 2i + LIF (2iL) medium for at least two passages were then dissociated with 0.25% TE, washed, centrifuged at 1200 rpm for 3 min, and resuspended in ES medium without 2iL. ESCs were then cultured in a new 6-well plate for 30 min to remove feeders based on differences in adherence to the dish, followed by seeding at a density of 10^5^ cells per well in HFN-coated 12-well plates. The differentiation medium containing the respective small-molecule compounds was changed every other day. After MACS for the removal of SSEA1^+^ cells, the remaining SSEA1^−^ cell population was considered GSCLCs and used for aggregation.

### Induction of meiosis

Induction of meiosis was achieved by aggregation of PGCs with E12.5_GSCs isolated from E12.5 female gonads, GSCLCs induced via V580_D6, ovary somatic cells isolated from 6 weeks old mice (P_6w_) and MEF cells. Aggregates were cultured for four days to induce meiosis in the wells of a low-cell-binding U-bottom 96-well Lipidure-Coat plate in gonad medium, containing M199 supplemented with 10% FBS, 1 mM L-glutamine, penicillin (100 U/mL), and streptomycin (100 μg/mL), 50 μg/mL Vc and 10 μM Rocki.

### Aggregation of P_6w_ via PHA

As P_6w_ cannot perfectly aggregate with PGCs, before adding the aggregate into a low-cell-binding U-bottom 96-well Lipidure-Coat plate, cells were resuspended in 100 μL of gonad medium. After the addition of phytohemagglutinin-P (PHA), cell suspensions were incubated at 37 °C for 10 min. Subsequently, the suspensions were centrifuged twice at 9000×*g* for 1 min to obtain re-aggregated pellets. The re-aggregated cells were gently picked with a truncated 200 μL micropipette, placed into a low-cell-binding U-bottom 96-well Lipidure-Coat plate filled with 200 μL of gonad medium, and cultured at 37 °C.

## Immunofluorescence microscopy of rOvaries and differentiated cells

rOvaries cultured on Transwell membranes were fixed for 1 h in 3.7% paraformaldehyde at 4 °C, dehydrated through 30% sucrose, and embedded in OCT (Optimal cutting temperature compound). After washing in PBS for 10 min, sections were fixed with ice acetone for 15 min at room temperature, subjected to 0.1% Triton X-100 for 30 min, blocked with 3% BSA in PBS for 2 h at room temperature or overnight at 4 °C, and then incubated with the primary antibodies against Foxl2 (ab5096, Abcam), GFP(ab183735, Abcam), Gata4 (sc-25310, Santa Cruz) or Vasa (ab13840, Abcam) overnight at 4 °C, washed and incubated for 2 h with appropriate fluorescence-conjugated secondary antibodies (Donkey anti-goat IgG (H + L), Alexa Fluor 594, A-11058, Invitrogen; Donkey anti-Mouse IgG (H + L), Alexa Fluor 488, A-21202, Invitrogen; Donkey anti-rabbit IgG (H + L), Alexa Fluor 594, A-21207, Invitrogen; Donkey anti-mouse IgG (H + L), Alexa Fluor 594,A-21203, Invitrogen). Samples were washed thrice in PBS, counterstained with 0.5 mg/mL Hoechst 33342 (H1398, MP) in Vectashield (VectorLabs) mounting medium. Fluorescence was detected and imaged using Axio-Imager Z2 Fluorescence Microscope (Zeiss).

Differentiated cells were immunostained by washing twice with PBS; then fixed in freshly prepared 3.7% paraformaldehyde in PBS (pH 7.4), permeabilized in 0.1% Triton X-100 (Sigma–Aldrich, Saint Louis, MO) in blocking solution (3% BSA in PBS) for 30 min, and incubated in blocking solution for 2 h. Cells were then incubated overnight at 4 °C with primary antibodies and secondary antibodies as described above. Nuclei were counterstained with 0.5 μg/mL Hoechst 33342 in Vectashield mounting medium. Fluorescence was imaged as previously described.

### Immunofluorescence microscopy of meiocyte spreads

Surface spreading of meiocytes was prepared by a drying-down technique and stained for synaptonemal complexes [45]. rOvaries were collected, digested in 0.05% TE for 10 min at 37 °C. Cell suspensions were intermingled with five volumes of MEF medium, centrifuged at 1200 rpm for 3 min and resuspended in 100 mM sucrose. The cell suspension was spread onto glass slides by dipping onto a thin layer of fixative (1% paraformaldehyde, 0.15% Triton X-100, pH = 9.2), which were maintained for at least 3 h in a humidified box and dried for 20 min at room temperature. The slides were then washed in water containing 0.4% Photo-flow (Kodak), and completely dried at room temperature. Dried slides were washed with 0.1% Triton X-100/PBS (PBST) for 10 min, and incubated with Blocking solution (ADB, 3% BSA, 2% goat serum/PBST) for 2 h at room temperature. Spreads were then incubated with anti-Sycp1 (ab15090, Abcam), anti-Sycp3 (NB300-230, Novus) antibody in ADB at 4 °C overnight, washed thrice, incubated with appropriate secondary antibodies, washed, and counterstained with 0.5 μg/mL Hoechst33342 in Vectashield mounting medium. Immunofluorescence was detected using an Axio-Imager Z2 Fluorescence Microscope.

### Western blot

Cells were washed at least twice in PBS and lysed in NP40 lysis buffer containing PMSF and cocktails on ice for 30 min, and then sonicated for 2 min at 60 amp at 2 s intervals. The concentration of the protein sample was measured by bicinchoninic acid and boiled in SDS sample buffer at 95 °C for 10 min. Next, 3 μg of protein were electrophoresed using 10% SDS-PAGE (Bio-Rad) and transferred to polyvinylidene fluoride membranes (PVDF, Millipore) using the Mini Trans-Blot system (Bio-Rad). Nonspecific binding was blocked in 5% skim milk in TBST at room temperature for 2 h or 4 °C overnight. Blots were then probed with primary antibodies Foxl2 (ab246511, Abcam), Nanog (ab80892, Abcam), Oct4 (sc5279, Santa Cruz), Gata4 (sc25310, Santa Cruz), and β-actin (P30002, Abmart) served as a loading control. Immunoreactivity bands were then probed for 2 h at room temperature with the appropriate secondary antibodies, HRP-goat anti-rabbit IgG or HRP-goat anti-mouse IgG(H + L). Protein bands were detected by Chemiluminescent HRP substrate (WBKLS0500, Millipore).

### Flow cytometry

V580_D6 were digested into single cells and stained with CD63-PE (143903, BioLegend) antibody for 20 min at 4 °C and washed with fluorescence-activated cell sorting (FACS) buffer, centrifuged at 220×*g* for 3 min, resuspended with FACS buffer, and then filtered by 70 μm flow tube before sorting. Flow cytometry analysis and sorting were performed on a BD FACSAria Fusion (BD Biosciences).

### RNA-sequencing (RNA-Seq)

Cells were harvested and total RNA extracted using RNeasy Mini kit (Qiagen), according to the manufacturers’ instruction, including a DNAse digestion. A total amount of 3 µg RNA per sample was used as input material for the RNA sample preparations. Sequencing libraries were generated using NEBNext® UltraTM RNA Library Prep Kit for Illumina® (NEB, USA) following manufacturer’s recommendations and index codes were added to attribute sequences to each sample. To select 250–300 bp cDNA fragments, the library fragments were purified with AMPure XP system (Beckman Coulter, Beverly, USA). Then 3 µL of USER Enzyme (NEB, USA) was used with size-selected, adaptor-ligated cDNA at 37 °C for 15 min followed by 5 min at 95 °C. PCR was performed with Phusion High-Fidelity DNA polymerase, Universal PCR primers and index (X) Primer. PCR products were purified (AMPure XP system) and library quality was assessed on the Agilent Bioanalyzer 2100 system and the index-coded samples was performed on a cBot Cluster Generation System using TruSeq PE Cluster Kit (Illumina) according to the manufacturer’s instructions. After cluster generation, the library preparations were sequenced on an Illumina Hiseq platform.

### Bioinformatics analysis

The clean reads were mapped to the Mus musculus mm10 reference genome (downloaded from http://genome.ucsc.edu/). Index of the reference genome was built using Hisat2 and paired-end clean reads were aligned to the reference genome using Hisat2 [46] with default parameters.

Reads were assigned and counted to genes using the featurecounts [47]. The resulting matrix of read counts was loaded into RStudio (R version 3.4.2), and DESeq2 [48] were used to identify DEGs. The resulting *P* values were adjusted using the Benjamini and Hochberg’s approach for controlling the false discovery rate. Genes identified by DESeq2 with an adjusted *P* value < 0.05 were assigned as differentially expressed. GO enrichment analysis of DEGs was implemented by the clusterProfiler R package [49] and DAVID (https://david.ncifcrf.gov/), in which gene length bias was corrected. GO terms with corrected *P* value < 0.05 were considered significantly enriched by DEGs. Additionally, the KEGG database was used to identify high-level functions and pathways associated with the DEGs (http://www.genome.jp/kegg/). Bar plots were drawn using ggpubr and ggplot.

### Single-cell library preparation and sequencing

A single-cell library was prepared using the 10 × Genomics Chromium Single Cell 3′ Library and Gel Bead Kit v2 (10 × Genomics, Pleasanton, CA, USA, 120237) according to the manufacturer’s instructions. To determine whether the cells obtained were viable (cell viability > 80%, cell concentrations = 1000 cells/μL) for downstream analysis, the cell viability was evaluated using trypan blue staining with a hemocytometer (Bio-Rad, Hercules, CA, USA, TC20) and the cell concentration was adjusted to 1000 cells/μL before loading to the single-cell chip. The Gel Bead in Emulsions (GEMs) were generated with the Chromium 10 × Single Cell System (10 × Genomics). To barcode cDNA in each cell, the cells were then lysed and reverse transcribed. cDNA recovery was performed using DynaBeads MyOne Silane Beads (Invitrogen, Carlsbad, CA, USA, 37002D) according to the manufacturer’s instructions. cDNA libraries were then prepared using 10 × Genomics Chromium Single Cell 3′ Library and Gel Bead Kit v2 following the manufacturer’s guide and sequencing was performed with an Illumina HiSeq X Ten sequencer (Illumina, San Diego, CA, USA) with pair-end 150 bp (PE150) reads.

### 10 × Genomics computational analysis

The Cell Ranger software suite was obtained from 10 × Genomics. Raw sequencing data was demultiplexed by Illumina bcl2fastq software to generate separate paired end read files for each sample, which were quality-checked using FastQC software. The Cell Ranger “count” script was used to align mouse fastq files to the mouse mm10 reference genome (Ensembl). The raw count matrices were imported into R for further processing. R studio (https://www.rstudio.com/) was used to run R scripts to perform hierarchical clustering and PCA. To identify distinct cell populations of the V580_D6 GSCLCs and E12.5_GSCs, cell clustering was performed using R software package Seurat 3.0 [50]. The count matrix was first normalized by library size and log transformed by Seurat. Transcriptomes with < 200 expressed genes and lowly expressed in three cells were discarded, cells with mitochondrial genes occupying > 40% of reads were defined as low-quality cells and filtered out. Two datasets of V580_D6 GSCLCs and E12.5_GSCs [51](GSE128553) were integrated by “IntegratedData” function of Seurat according to instructions. Uniform Manifold Approximation (U-MAP) was used for visualization and clustering. The “FindConservedMarkers” function was used to identify canonical cell-type marker genes that are conserved across conditions.

### Statistical analysis

Data were analyzed using ANOVA with Fisher’s protected least-significant difference (PLSD) using the StatView software from SAS Institute Inc. (Cary, NC), two-tailed Student’s *t* test, or *χ*^2^ test or Wilcoxon–Mann–Whitney rank sum test dependent on specific experiments. A *P* value < 0.05 was considered statistically significant.

## Results

### Transcriptome features at different stages of gonadal somatic cell development

**Fig. 1:**
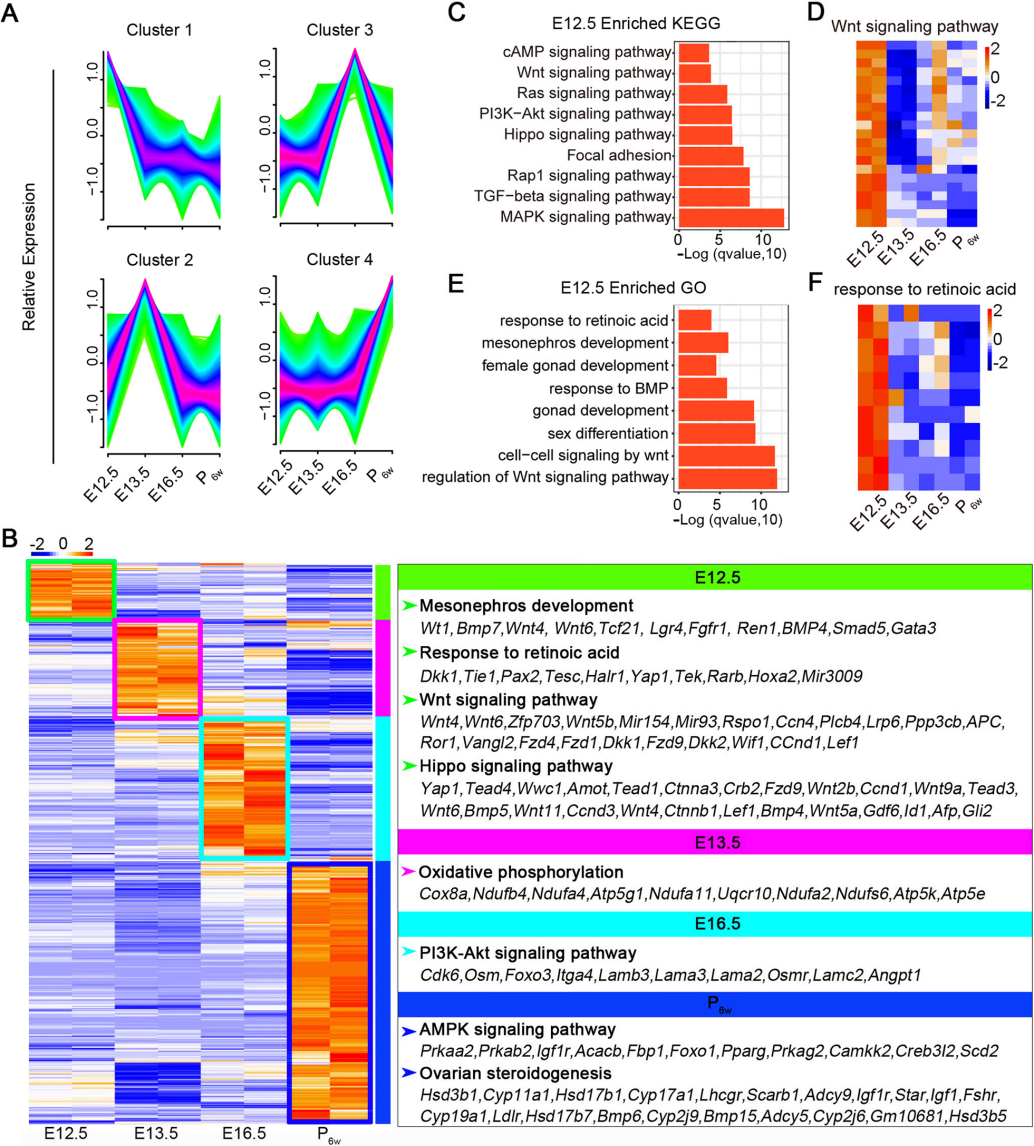
Transcriptome of fetal and adult gonadal somatic cells. **A** Time-course line chart showing changes in gene expression during embryonic development. Cluster 1, 2, 3, and 4 represent E12.5, E13.5, E16.5, and P_6w_, respectively. **B** Enriched terms in each stage. Genes upregulated in E12.5_GSCs were clustered in mesonephric development, response to retinoic acid, Wnt signaling pathway, and Hippo signaling pathways. **C** KEGG analysis of enriched genes in E12.5_GSCs. Wnt, Hippo, cAMP signaling pathway were clustered in E12.5 stage. **D** Pheatmap showing genes enriched in Wnt signaling pathway. **E** GO analysis of enriched genes in E12.5_GSCs, response to retinoic acid, mesonephric development, Wnt-related signaling pathway were clustered in E12.5_GSCs. **F** Pheatmap showing genes enriched in response to retinoic acid

We performed bulk RNA-sequencing (RNA-seq) of gonadal somatic cells from mice at different developmental stages (E12.5, E13.5, E16.5, and P_6w_). Principal component analysis (PCA) indicated that the two samples had better reproducibility during the same period (Additional file 1: Fig. S1A). Correlation between E12.5 and E13.5 was as high as 0.95 or 0.96 (Additional file 1: Fig. S1B). Differential expression analysis revealed more than 2000 differentially expressed genes (DEGs) between E13.5 and E12.5, approximately 6000 DEGs between E16.5 and E12.5, and nearly 8000 DEGs between P_6w_ and E12.5 (Additional file 1: Fig. S1C). A time-course line chart shows changes in gene expression during embryonic development (Fig. 1A). We analyzed DEGs among the developmental stages. *Foxl2* was gradually upregulated during gonadal development (Additional file 1: Fig. S1D), which was also confirmed by the qPCR result (Additional file 1: Fig. S1E). Pairwise and multi-group DEG analysis of the four stages revealed that genes that were specifically and highly expressed in E12.5 were enriched for mesonephric development, response to retinoic acid (RA), and the Wnt and Hippo signaling pathways. Genes upregulated in E13.5 were enriched for oxidative phosphorylation, and those in E16.5 were enriched for the PI3K-Akt signaling pathway, whereas those upregulated in P_6w_ were primarily enriched for the AMPK signaling pathway and ovarian steroidogenesis (Fig. 1B). As we focused on E12.5, we analyzed Gene Ontology (GO) terms and Kyoto Encyclopedia of Genes and Genomes (KEGG) signaling pathways enriched at this stage (Fig. 1C–F), revealing characteristic expression pattern at each stage.

**Fig. 2:**
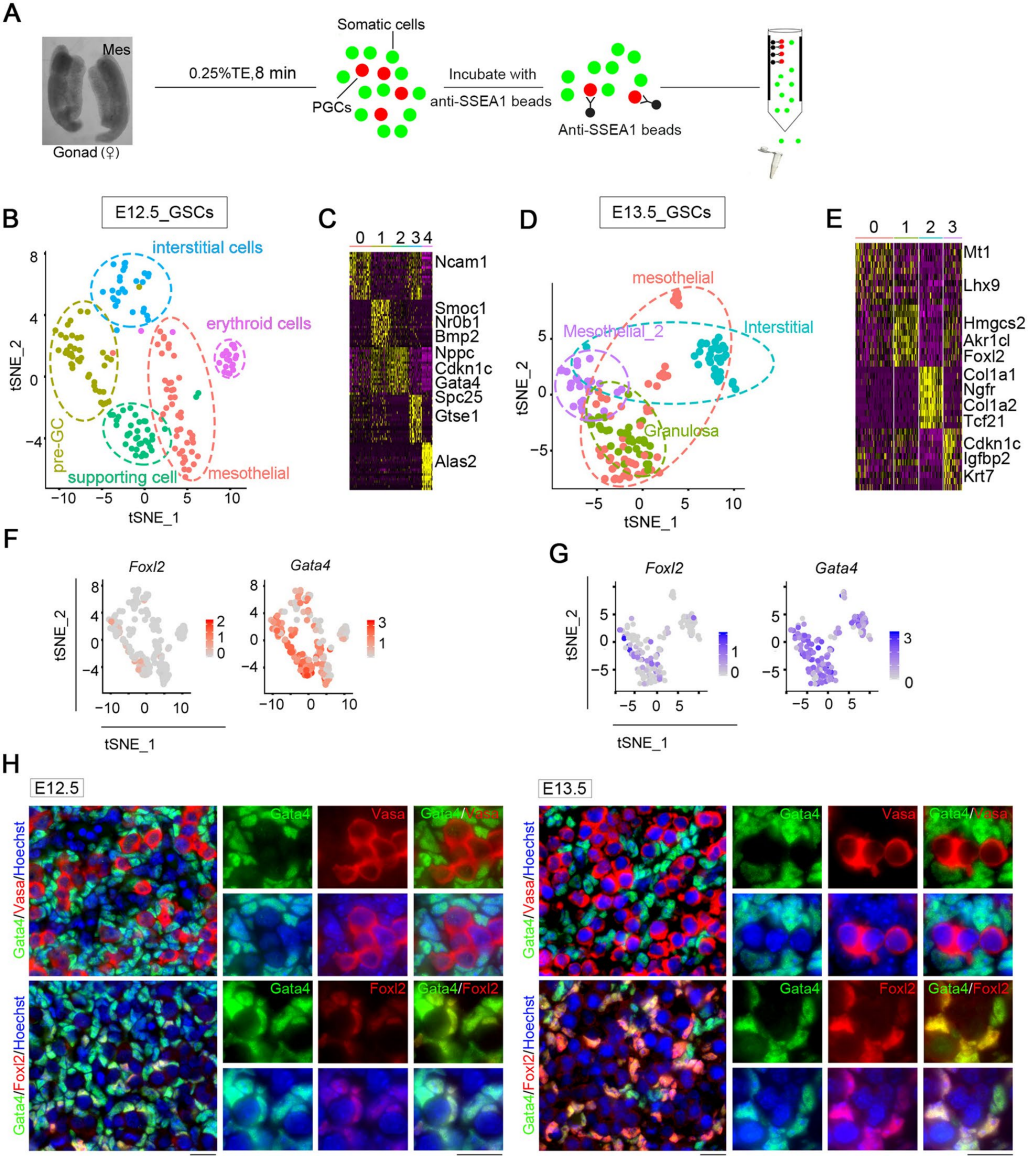
Single cell analysis of E12.5_ and E13.5_ GSCs. **A** Schematic of PGCs and somatic cells separation. Mes: mesonephros. **B** tSNE plot showing the five clusters distribution of E12.5_GSCs. **C** Pheatmap showing marker genes of each cluster in E12.5_GSCs. **D** tSNE plot showing the four clusters distribution of E13.5_GSCs. **E** Pheatmap showing marker genes of each cluster in E13.5_GSCs. **F** Distribution and expression of marker genes including *Foxl2* and *Gata4* in E12.5_GSCs. **G** Distribution and expression of marker genes including *Foxl2* and *Gata4* in E13.5_GSCs. **H** Distribution of Gata4, Vasa and Foxl2 in E12.5 and E13.5 gonads. Scale bar = 20 μm

To further determine the gene expression landscape and dissect the cellular heterogeneity in the initiation of meiosis of female germ cells, we dissociated gonads from E12.5 and E13.5 embryos using the magnetic-activated cell sorting (MACS) and prepared somatic single-cell suspensions for smart-seq2 single-cell analysis (Fig. 2A). After filtering low-quality cells based on the number of genes and percentage of mitochondrial genes, we obtained a total of 345 GSCs (172 cells for E12.5 and 173 cells for E13.5), with the median number of genes per cell in the range 6000–8000. We next performed t-distributed stochastic neighbor embedding (tSNE) clustering analysis to dissect cellular heterogeneity within the somatic cell populations. After tSNE projection, five and four clusters of E12.5 and E13.5 somatic cells were separated via Seurat (Fig. 2B–E), and the pheatmap revealed marker genes of each cluster. In E12.5_GSCs, cluster 0 formed the early progenitor cell population and expressed marker, *Nr2f1*; cluster 1 represented pre-granulosa cells expressing *Foxl2* and *Bmp2*; cluster 2 expressed the supporting cell marker *Amhr2*; cluster 3 expressed *Bgn*, a marker of interstitial cells; and cluster 4 expressed erythroid cell marker *Alas*. In E13.5_GSCs, cluster 0 expressed the mesothelial cell markers *Lhx9*, clusters 1 expressed the granulosa cell marker *Foxl2*, whereas cluster 2 expressed the fetal Leydig cell progenitor marker *Tcf21*.

To identify somatic populations among E12.5_GSCs and E13.5_GSCs, we assessed *Foxl2* and *Gata4* expression, and observed that the number of *Foxl2*-expressing cells increased with embryonic development, whereas that of *Gata4*-expressing cells remained high throughout (Fig. 2F, G). To further verify the distribution of these cells in gonads, we analyzed *Foxl2* expression using qPCR and performed immunofluorescence (IF) staining for Gata4, Foxl2, and Vasa, a germ cell marker (Fig. 2H). Foxl2-positive cells surrounded germ cells, with Foxl2 expression observed in Gata4-positive cells. Based on the mRNA level and protein levels, we concluded that Foxl2-positive cells originated from Gata4-positive cells, and *Foxl2* was gradually upregulated during embryonic gonadal development.

### GSCLCs induction from ESCs by small-molecule compounds

**Fig. 3:**
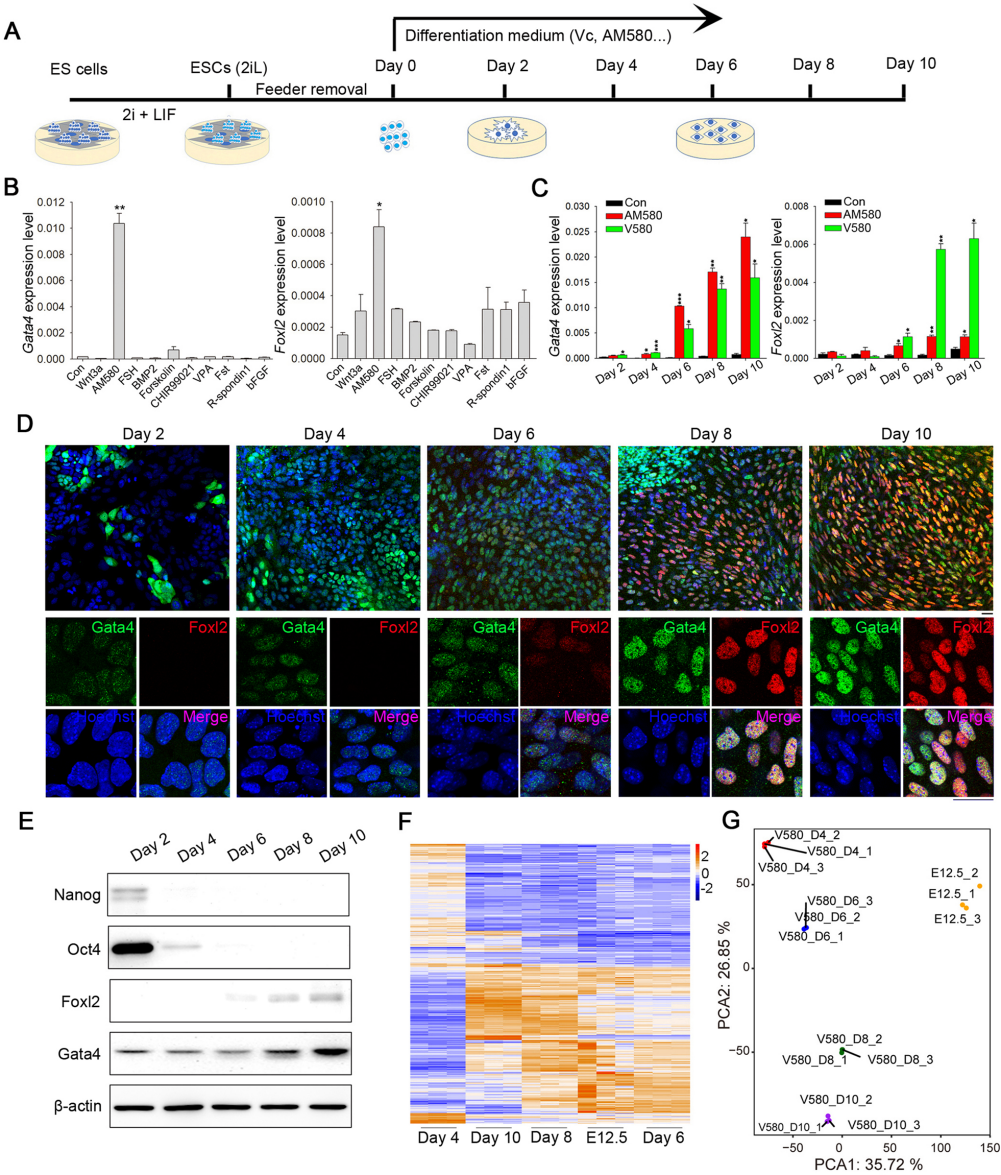
Induction of E12.5_GSCLCs by small molecules. **A** Schematic illustration of in vitro chemical induction strategy. **B** qPCR detection of *Gata4* and *Foxl2* expression in GSCLCs induced by treatment with different small-molecule compounds. Bars = Mean ± SEM (*n* = 3). *P < 0.05; **P < 0.01; ***P < 0.001. **C** qPCR detection of *Gata4* and *Foxl2* expression in GSCLCs induced by AM580 and V580. Bars = Mean ± SEM (*n* = 3). *P < 0.05; **P < 0.01; ***P < 0.001. **D** Immunofluorescence staining of Gata4 (green) and Foxl2 (red) in GSCLCs induced by V580. Scale bar = 20 μm. **E** Protein levels of pluripotency markers (Nanog and Oct4) and E12.5_GSCs markers (Gata4 and Foxl2) in GSCLCs induced by V580 were determined via western blot analysis. β-actin served as a loading control. **F** Heatmap highlighting DEGs compared with E12.5_GSCs, determined using RNA-seq. **G** PCA shows that V580_D6 GSCLCs and E12.5_GSCs were closer with regard to the overall transcriptome

Based on the unique transcriptome profile of E12.5_GSCs, we sought to establish a new approach for differentiating ESCs into E12.5_GSCLCs through treatment with small-molecule compounds (Fig. 3A). The ESCs used in this study were female pluripotent stem cells expressing (or w/o) β-actin-green fluorescent protein (GFP) generated in our laboratory and stably maintained on inactivated MEF [44, 52]. Pluripotency of these ESCs was evidenced by strong competence for generation of chimeras that gave rise to germline-competent offspring. Through the first round of small-molecule compound screening, we identified AM580 as a strong candidate, which induced upregulation of the E12.5_GSCs markers *Gata4* and *Foxl2* (Fig. 3B). A progressive increase in the expression of both markers was observed following AM580 treatment, whereas the expression of pluripotent genes, such as *Nanog* and *Oct4*, declined on approximately day 2 and abruptly decreased thereafter (Additional file 1: Fig. S2A, B). In addition, we used an ESC line without GFP for western blot and IF analysis, which validated the qPCR results (Additional file 1: Fig. S2C–E).

We next attempted to optimize our protocol through identification of additional small-molecule compounds and found that the addition of 50 μg/mL Vc during differentiation facilitated somatic cell induction compared with addition of AM580 alone (Fig. 3C, D and Additional file 1: Fig. S2D), which was confirmed at the protein level using western blot (Fig. 3E), whereas IF analysis indicated that the number of Foxl2-positive cells was obviously increased (Fig. 3D). In line with the established gradual increase in *Foxl2* expression during gonad development, our results indicated that *Gata4* and *Foxl2* were gradually upregulated throughout the induction process (Fig, 3C and Additional file 1: Fig. S2A). We then investigated whether the expression profile at a certain differentiation stage resembles that of E12.5_GSCs. We performed RNA-seq analysis of cell populations from different days of V580 induction. The transcriptome of V580_D6 GSCLCs was similar to that of E12.5_GSCs (Fig. 3F, G). Correlation analysis suggested that the similarity of the overall transcriptome between V580_D6 GSCLCs and E12.5_GSCs could reach 70.29% (Additional file 1: Fig. S3A). Further, the morphology of day 6 cells was similar to that of E12.5_GSCs (Additional file 1: Fig. S3B, C). Treatment with Vc alone did not induce ESCs into GSCLCs. The morphology and marker expression levels in cells treated with Vc alone were similar to those of controls, with no GSCLCs observed even at day 10 (Additional file 1: Fig. S3D-G).

### Induction of PGC entry into meiosis by V580_D6 GSCLCs

**Fig. 4:**
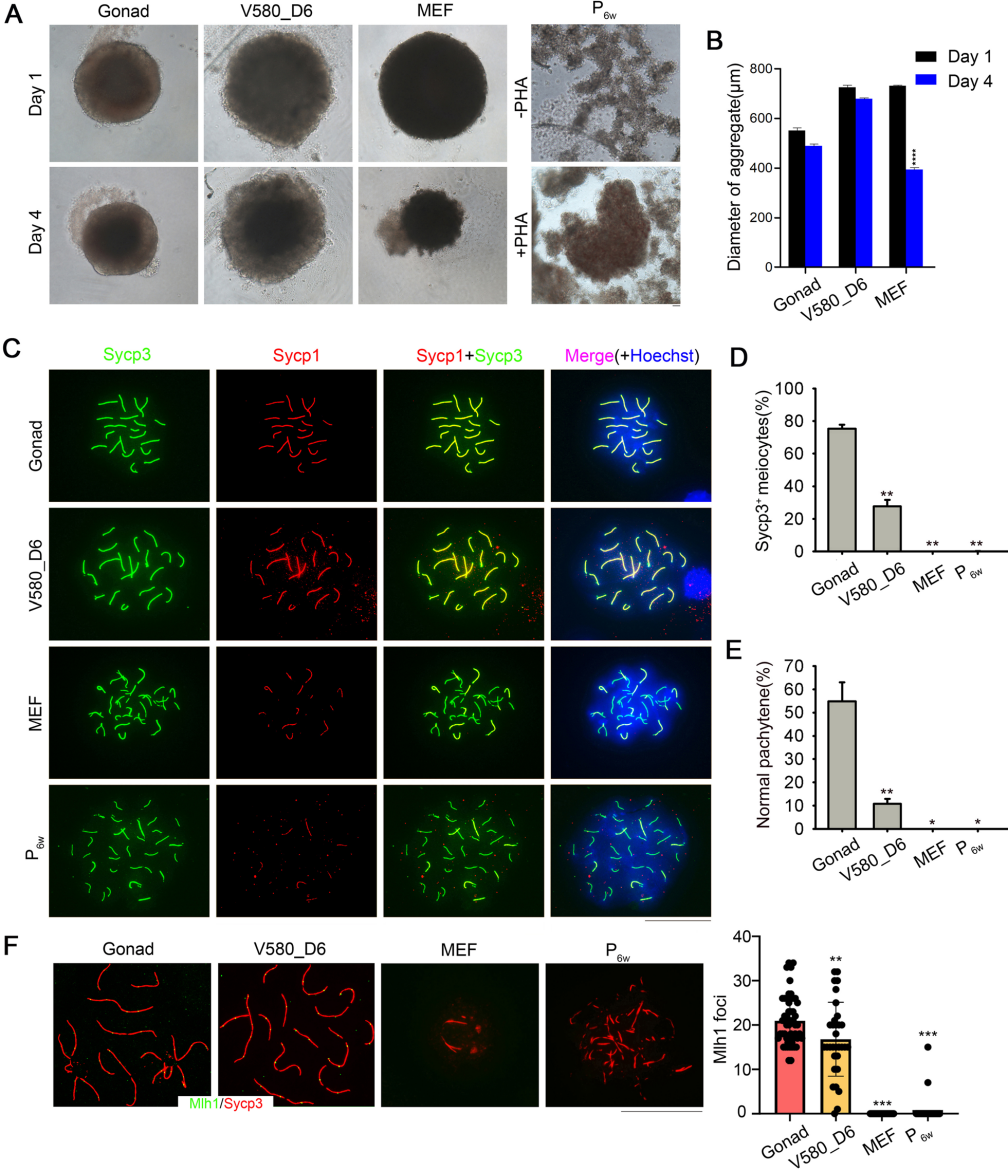
V580_D6 GSCLCs, but not MEF and P_6w_, promote meiosis. **A** Morphology of different cell types after aggregating with PGCs. Scale bar = 50 μm. **B** The diameter of aggregates. The size of MEF_aggregates was obviously smaller, and P_6w__aggregates did not form round structures even with PHA treatment, whereas Gonad_aggregates and V580_D6 aggregates did not change considerably in size. Bars = Mean ± SEM (*n* = 10). *P < 0.05; **P < 0.01; ***P < 0.001. **C** E12.5 female gonad reaggregation supports normal meiosis initiation in PGCs; V580_D6 GSCLCs can also support the normal meiosis of PGCs. In aggregates of MEF or P_6w_ with PGCs, only a small amount of Sycp3 expression was observed, and the expression of Sycp1 was inhibited or completely suppressed in some cases, resulting in the inability of PGCs to proceed with normal meiosis. Scale bar = 20 μm. **D** Proportion of PGCs that can initiate meiosis in the different groups. Sycp3^+^ cells could not be detected in MEF_ and P_6w__ aggregates. Bars = Mean ± SEM (*n* = 10). *P < 0.05; **P < 0.01; ***P < 0.001. **E** Percentage of normal synaptonemal complexes in different groups based on pachytene spread (*n* = 40). Bars = Mean ± SEM (n = 10). *P < 0.05; **P < 0.01; ***P < 0.001. **F** Statistics of Mlh1 foci per cell at the pachytene stage. No Mlh1 foci formed in MEF_ and P_6w__ aggregates. Bars = Mean ± SEM (n ≥ 10). *P < 0.05; **P < 0.01; ***P < 0.001. Scale bar = 20 μm

Abnormal meiosis can trigger infertility, premature ovarian failure, and genetic diseases [10, 53]. This was recently confirmed by reconstituting the oocyte transcriptional network; oocytes obtained in this manner exhibited abnormal chromosomal configuration due to the absence of meiosis [54]. To determine whether V580_D6 GSCLCs support the entry of PGCs into meiosis, we aggregated V580_D6 GSCLCs with PGCs isolated from E12.5 gonads to form aggregates (V580_D6 aggregates), which were then compared to aggregates with MEF (MEF_aggregates), P_6w_ (P_6w__aggregates), or E12.5_GSCs (Gonad_ aggregates), as controls. The size of MEF_aggregates on day 4 was obviously smaller than day 1, while P_6w__aggregates did not form round structures even with PHA treatment; Gonad_ and V580_D6 aggregates did not change considerably in size (Fig. 4A, B).

To determine the normality of meiosis progression in PGCs from different aggregates, we analyzed homologous chromosome pairing and synapsis via IF analysis of Sycp1/3 elements. Through co-staining of the Sycp1 and Sycp3 elements, normal meiosis progression was detected in V580_D6 and Gonad_ aggregates, whereas no normal pachytene meiocytes were detected in MEF_ and P_6w__ aggregates (Fig. 4C). Furthermore, Sycp3-positive (Sycp3^+^) cells were not detected in MEF_ and P_6w__ aggregates, thus confirming that the maturation of PGCs requires developmentally matched gonadal somatic cells. Twenty-seven percent of PGCs in V580_D6 aggregates differentiated into Sycp3^+^ meiocytes, which was lower than that observed in E12.5_aggregates (Fig. 4D). As the other control groups were highly atrophic with no normal meiocytes in pachytene, we did not determine their proportion of meiocytes. Sycp1 and Sycp3 formed the axial elements of the synaptonemal complex completed at the pachytene stage, and synaptonemal complex elements were detected in V580_D6 aggregates (Fig. 4E). In addition, the cell population induced by Vc or AM580 treatment alone did not initiate normal meiosis (Additional file 1: Fig. S4A, B). This was also confirmed by the fact that V580_D6 GSCLCs exhibited certain E12.5_GSCs’ function. Mlh1, a marker of meiotic recombination, was also detected in V580_D6 aggregates (Fig. 4F). As Wnt4 and R-spondin1 play significant roles in ovary development, their absence leads to partial sex reversal [55–58]. We designed three groups to assess the function of both factors in GSCLCs’ induction. First, we determined a concentration suitable for Wnt signaling pathway activation (Additional file 1: Fig. S4C) and aggregated with E12.5_PGCs (Additional file 1: Fig. S4D). No Sycp1/Sycp3 was detected in these aggregates (Additional file 1: Fig. S4D).

To determine the pathways underlying V580_D6 GSCLCs-stimulated meiosis initiation, we compared expression profiles between V580_D6 GSCLCs and E12.5_GSCs and delineated the genes and pathways altered in V580_D6 GSCLCs. The heatmap indicated that genes related to key pathways in E12.5_GSCs exhibited a similar expression pattern as in V580_D6 GSCLCs, however, at a lower level in MEF and P_6w_ (Additional file 1: Fig. S5A). PCA also confirmed that V580_D6 GSCLCs resembled E12.5_GSCs (Additional file 1: Fig. S5B). KEGG analysis indicated that genes essential for E12.5_GSCs development, such as those involved in the Wnt and Hippo signaling pathways, were upregulated in V580_D6 GSCLCs (Additional file 1: Fig. S5C). GO analysis also revealed a significant enrichment of genes for regulation of Wnt signaling, cellular response to retinoic acid, mesonephric development, and reproductive structure development (Additional file 1: Fig. S5D). Representative genes upregulated for cellular response to retinoic acid and the Hippo signaling pathway are listed (Additional file 1: Fig. S5E). In P_6w_, Wnt signaling, Hippo signaling pathway, mesonephric development, and gland development were upregulated to a lesser extent, whereas genes enriched for the AMPK signaling pathway, ovarian steroidogenesis, and apoptosis were highly expressed (Additional file 1: Fig. S6A-E). In MEF, the Wnt signaling pathway and steroid biosynthesis, which are indispensable in E12.5_GSCs, were not activated, whereas apoptosis-related pathways, such as the p53 signaling pathway, were activated (Additional file 1: Fig. S6F-K). To further elucidate the underlying molecular mechanisms, we performed RNA-seq analysis of the transcriptome for control GSCLCs at day 0 as well as GSCLCs treated with Vc, AM580, or V580 for 24 h and 48 h. Compared with those in controls, after 24 h of induction, only 99 genes were upregulated and 76 were downregulated in the Vc-induced cell population; 259 were upregulated and 62 were downregulated in the AM580-induced cell population; 223 were downregulated and 410 were upregulated in the V580-induced cell population (Additional file 1: Fig. S7A, *P* value < 0.05, fold change ≥ 2). After 48 h of induction, 84 genes were upregulated and 90 were downregulated in Vc-induced cells; 480 were downregulated and 1180 were upregulated in AM580-induced cells; 609 were downregulated and 1194 were upregulated in V580-induced cells (Additional file 1: Fig. S7A). KEGG analysis indicated that genes enriched for Wnt signaling, reproductive system development, hormone metabolic process, and cellular response to retinoic acid, which are essential pathways for GSCs development, were upregulated after V580 stimulation for 48 h, which laid the foundation for their later differentiation into GSCLCs (Additional file 1: Fig. S7B). Genes enriched for canonical Wnt signaling and response to retinoic acid are listed (Additional file 1: Fig. S7C, D).

### Resemblance of CD63^+^_GSCLCs to E12.5_GSCs

**Fig. 5:**
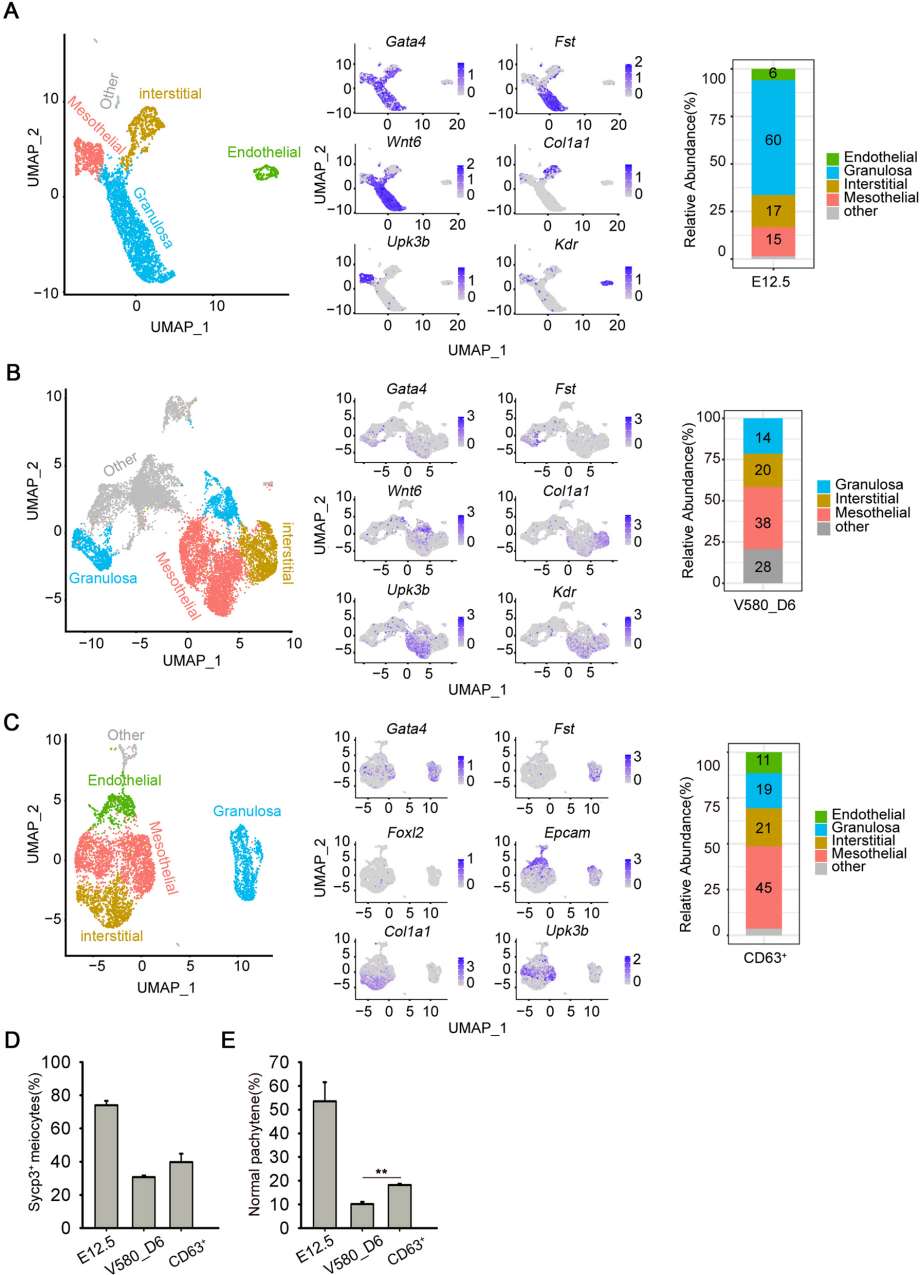
Cell types in the induced GSCLCs revealed by single-cell RNA-seq analysis. **A** Left: U-map showing the distribution of E12.5_GSCs. Middle: Marker genes are indicated by color; expression gradually increases from gray to blue. Right: Proportion of each cell cluster at E12.5. **B** Left: U-map showing the distribution of V580_D6 GSCLCs. Middle: Marker genes are indicated by color; expression gradually increases with from gray to blue. Right: Proportion of each cell cluster among V580_D6 GSCLCs. **C** Left: U-map showing the distribution of CD63^+^_GSCLCs Middle: Marker genes are shown by the color; expression gradually increases from gray to blue. Right: Proportion of each cell cluster among CD63^+^_GSCLCs. **D** Percentage of PGCs that can initiate meiosis in the CD63^+^ group was slightly upregulated. **E** Percentage of synaptonemal complexes in the CD63^+^ group based on pachytene spread (*n* = 40) was significantly upregulated. Bars = Mean ± SEM (*n* = 10). *P < 0.05; **P < 0.01; ***P < 0.001

To determine the cell type present among V580_D6 GSCLCs, we performed 10 × Genomics sequencing in triplicate. We compared the V580_D6 GSCLCs single-cell data with the previously published 10 × sequencing data from E12.5 gonads (including germ cells and gonadal cells) [51]. Seurat was used to integrate the two datasets together for unified analysis. The mesothelial cells, endothelial cells, interstitial cells, and granulosa cells present in E12.5_GSCs were also among the V580_D6 GSCLCs. The proportions of endothelial cells and mesenchymal cells were similar to those in vivo, however, that of precursor granulosa cells was relatively low, accounting for only 14%, compared with values of up to 60% in E12.5_GSCs. In addition, the proportion of unknown cell types was as high as 28% (Fig. 5A, B).

Following filtering based on CD63 expression (CD63^+^_GSCLCs), the GSCLCs population resembled E12.5_GSCs to a greater extent. The various cell types present in E12.5_GSCs were observed among CD63^+^_GSCLCs, including 45% mesothelial cells, 11% endothelial cells, 21% interstitial cells, and 19% granulosa cells. The precursor granulosa cells increased from 14 to 19%, and the proportion of unknown cell types was greatly reduced (Fig. 5C). Aggregates with CD63^+^_GSCLCs (CD63^+^_aggregates) exhibited an increased proportion of PGCs initiating meiosis compared with V580_D6 aggregates (Fig. 5D). Proportion of PGCs in the pachytene stage was also significantly increased (Fig. 5E).

**Fig. 6:**
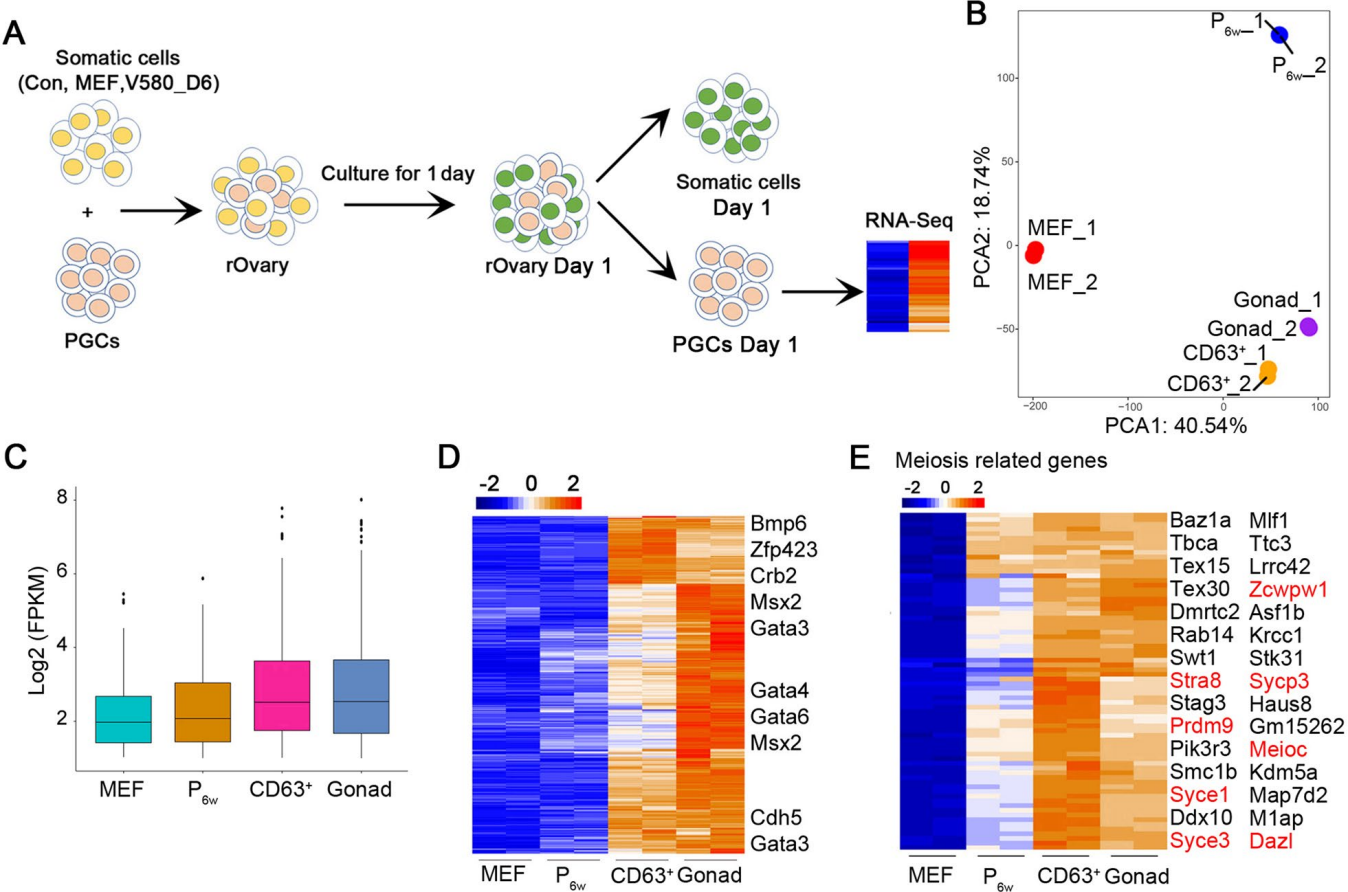
PGCs aggregated with CD63^+^_GSCLCs display transcriptome resembling PGCs aggregated with E12.5_GSCs. **A** Schematic illustration of PGC separation and collection. **B** PCA using all genes tested showing close relationship between CD63^+^ and gonad. **C** Boxplot showing global gene expression in CD63^+^, MEF, and P_6w_ compared with gonad by RNA-seq. **D** Heatmap highlighting the expression pattern of upregulated genes in CD63^+^, MEF, and P_6w_ compared with Gonad by RNA-seq. **E** Pheatmap showing expression of meiosis related genes in CD63^+^, MEF, P_6w_ and Gonad

To further confirm that the CD63^+^_GSCLCs indeed shared E12.5_GSCs’ function, we cultured the aggregates with gonad medium for 1 day, followed by separation using MACS columns and RNA-seq analysis of the PGCs transcriptome (Fig. 6A). PCA showed good repeatability and indicated that transcriptome of PGCs in Gonad_ and CD63^+^_ aggregates were similar (Fig. 6B). PGCs separated from Gonad_ and CD63^+^_ aggregates were readily distinguishable from MEF_ and P_6w__ aggregates based on global gene and specific marker expression (Fig. 6C, D). Numerous genes related to meiosis, including *Dazl* and *Stra8*, were upregulated in PGCs isolated from CD63^+^_ and Gonad_ aggregates (Fig. 6E).

### Vasa-positive (Vasa^+^) cells in follicles of Gonad_ and CD63^+^_ rOvaries

**Fig. 7:**
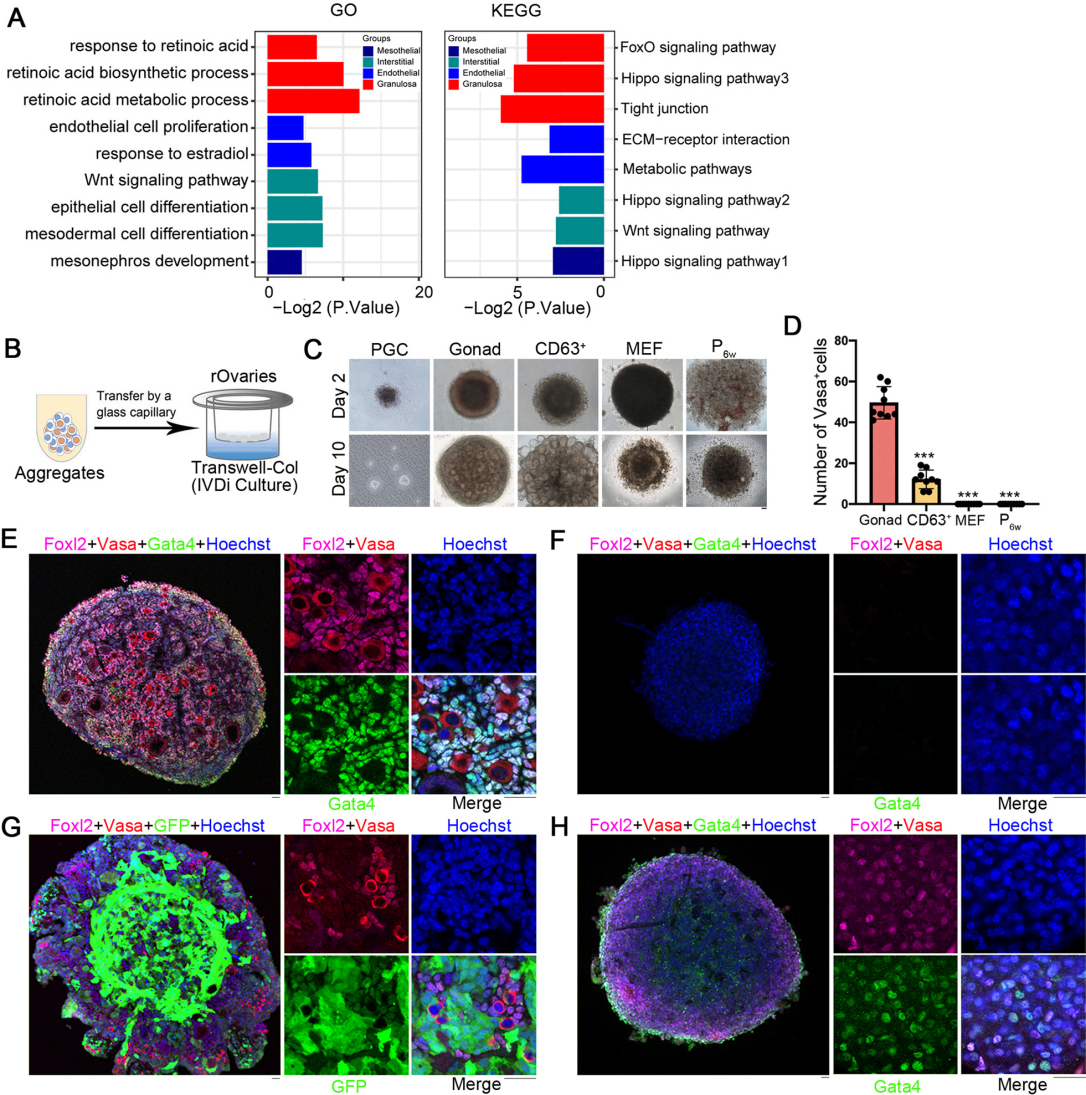
Vasa^+^ cells can be detected in CD63^+^_rOvaries cultured for 10 days. **A** GO and KEGG analysis of CD63^+^_GSCLCs. **B** Schematic of the IVDi culture. **C** Morphology of rOvaries in the culture system on day 2 and day 10. Scale bar = 50 μm. Pure PGC is scattered and nearly disappears on day 10. Scale bar = 50 μm. **D** Proportion of Vasa^+^ cells in different groups. Vasa^+^ cells can be detected in Gonad_ and CD63^+^_ rOvaries cultured for 10 days in vitro, but no such cells were observed in MEF_ and P_6w__ rOvaries. Bars = Mean ± SEM (*n* = 10). *P < 0.05; **P < 0.01; ***P < 0.001. **E** Distribution of Vasa, Foxl2, and Gata4 in E12.5_rOvaries. Scale bar = 20 μm. **F** Distribution of Vasa, Foxl2, and Gata4 in MEF_rOvaries. Scale bar = 20 μm. **G** Distribution of Vasa, Foxl2, and Gata4 in CD63^+^_rOvaries, GFP: CD63^+^_GSCLCs. Scale bar = 20 μm. **H** Distribution of Vasa, Foxl2, and Gata4 in P_6w__rOvaries. Scale bar = 20 μm

GO and KEGG analysis of CD63^+^_GSCLCs revealed enrichment of retinoic acid related pathways and Hippo signaling in granulosa cell populations and Wnt-related pathway enrichment in interstitial cell populations. In the endothelial cell population, pathways related to endothelial cell proliferation and response to estradiol were enriched, whereas pathways related to mesonephric development and Hippo signaling were enriched in the mesothelial population (Fig. 7A). These pathways are also activated in the E12.5_GSCs population.

We attempted to reconstruct the oogenesis in vitro [33]. In this method (Fig. 7B), the cell population was first cultured in a low-adhesion 96-well plate for 2 days to form the aggregates, which were then transferred onto Transwell membranes, followed by culture under in vitro differentiation (IVDi) conditions. The follicle-like structures formed by CD63^+^_rOvaries was similar to that formed by the reaggregation of E12.5 gonads in vivo (Fig. 7C). Immunofluorescence microscopy revealed Vasa^+^ cells in Gonad_ and CD63^+^_ rOvaries cultured for 10 days in vitro, however, no such cells were observed in MEF_ or P_6w__ rOvaries (Fig. 7D–H). In CD63^+^_rOvaries cultured for 21 days, we detected Vasa^+^ cells in later stage (Additional file 1: Fig. S8), further verifying that CD63^+^_GSCLCs indeed support the development of PGCs to form follicles. However, the efficiency was far lower than that of the normal developing follicles in rOvaries formed from E12.5_GSCs.

## Discussion

We have been able to generate GSCLCs from ESCs via small-molecule treatment compounds. Specifically, combined treatment with Vc and AM580 induced GSCLCs from ESCs. *Gata4* and *Foxl2* expression gradually increased throughout the induction process, in line with the established *Foxl2* upregulation during gonad development. Induction for short time is insufficient for achieving appropriate GSCLCs, yet prolonged induction tends to have more matured somatic cells. Through PCA and other analysis, we found that the transcriptome of V580_D6 GSCLCs resembled that of E12.5_GSCs and can support the initiation of meiosis in PGCs. Although V580_D6 GSCLCs did so to a relatively limited extent, control cells, MEF, and P_6w_ did not stimulate meiosis at all. In these groups, few PGCs exhibited Sycp3 expression, and Sycp1 was negligibly expressed. Moreover, V580_D6 GSCLCs partially exhibited E12.5_GSCs’ function. Whereas cell sorting for CD63 revealed high similarity between E12.5_GSCs and CD63^+^_GSCLCs, which promote PGCs to undergo follicuologenesis containing Vasa^+^ cells, suggesting that CD63^+^_GSCLCs and E12.5_GSCs engage in similar functions.

Mature oocytes with full potency were generated through culturing ESCs and induced pluripotent stem cells. Moreover, pluripotent stem cell lines were re-derived from the oocytes that were generated in vitro, thereby recapitulating the full female germline cycle in a dish [33]. However, all of these culture systems require matched somatic cells, which are obtained from embryos at E12.5. Yoshino et al. reported that fetal gonadal somatic-like cells can be induced from ESCs [41]. Our data complemented the findings of Yoshino et al., demonstrating that fetal gonadal somatic-like cells can be induced by only two small molecules from ESCs, which function to promote meiosis induction and progression. Nevertheless, considerable research is required before the induced cells can be made to exert the same function of E12.5_GSCs.

## Conclusion

In summary, our preliminary study demonstrates that fetal gonadal somatic-like cells can be induced by only two small molecules from ESCs, which function to promote meiosis induction and progression. We believe that our study adds contribution to the literature because the approach described will facilitate more in-depth studies of oocyte production as well as research into the potential treatment of female infertility.

## Supplementary Information

**Additional file 1**. Supplementary Figures and Legends. Supplementary Figures S1-S8. Supplementary Table S1.

## Data Availability

Relevant datasets have been uploaded as part of additional files. The accession numbers of RNA-seq data reported in this study is GEO: GSE181501. Data of E12.5_GSCs (10 × Genomics) were downloaded from NCBI [51]: GSE128553. The dataset used and/or analyzed during the current study are available from the corresponding author upon reasonable request.

## Abbreviations

ESCs: Embryonic stem cells
E12.5_GSCs: E12.5_gonadal somatic cells
PGCLCs: Primordial germ cell like cells
PGCs: Primordial germ cells
E12.5_GSCLCs: E12.5_GSC-like cells
