# Supplementary material for: Induction of meiosis by embryonic gonadal somatic cells differentiated from pluripotent stem cells
Source: Stem Cell Res Ther. 2021 Dec 20;12:607. doi: 10.1186/s13287-021-02672-4 (PMC8686525; doi:10.1186/s13287-021-02672-4)
Supplement: Supplementary file 1 — Additional file 1. Supplementary Figures and Legends. Supplementary Figures S1-S8. Supplementary Table S1. [file 13287_2021_2672_MOESM1_ESM.docx]

**Supporting Information:**

**Induction of meiosis by embryonic gonadal somatic cells differentiated from pluripotent stem cells**

**Wang, *et al.***

**The Following Files are Included:**

**Supplementary Figures and Legends**

**Supplementary Figures S1-S8**

**Supplementary Table S1**

**Supplementary Figures and Legends**


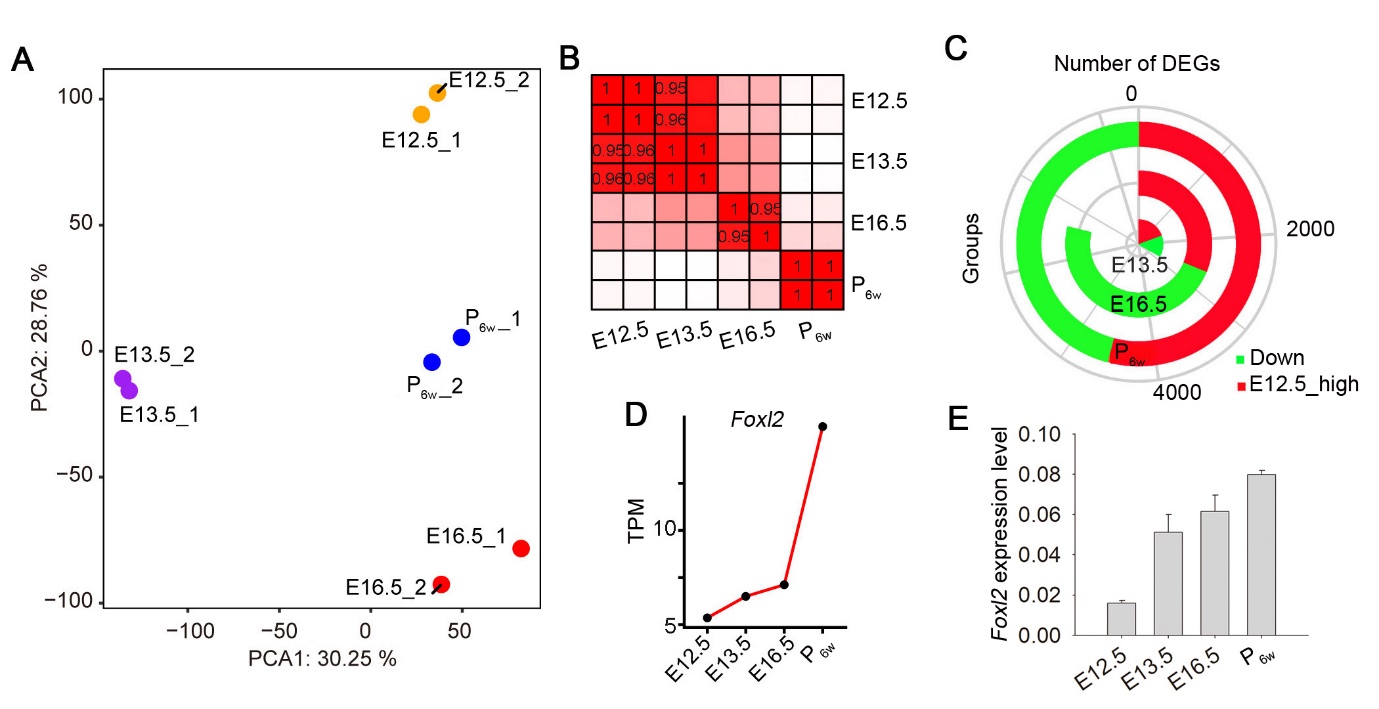


**Supplementary Figure** **S1. Transcriptome analysis of somatic cells at different stages of development.**

(A) PCA of E12.5, E13.5, E16.5, P_6w_ showing good repeatability.

(B) Pearson's correlation coefficient graph analysis of E12.5, E13.5, E16.5, and P_6w_. The value of 1.0 represents the total positive correlation, and 0 represents no correlation between two samples.

(C) Differential expression analysis showing the number of DEGs of E13.5, E16.5, and P_6w_ compared with E12.5. The number of DEGs were gradually increased with the development of gonads.

(D) Line chart showing expression level of *Foxl2* in each stage by RNA-seq data.

(E) *Foxl2* expression was gradually upregulated during embryonic gonad development.


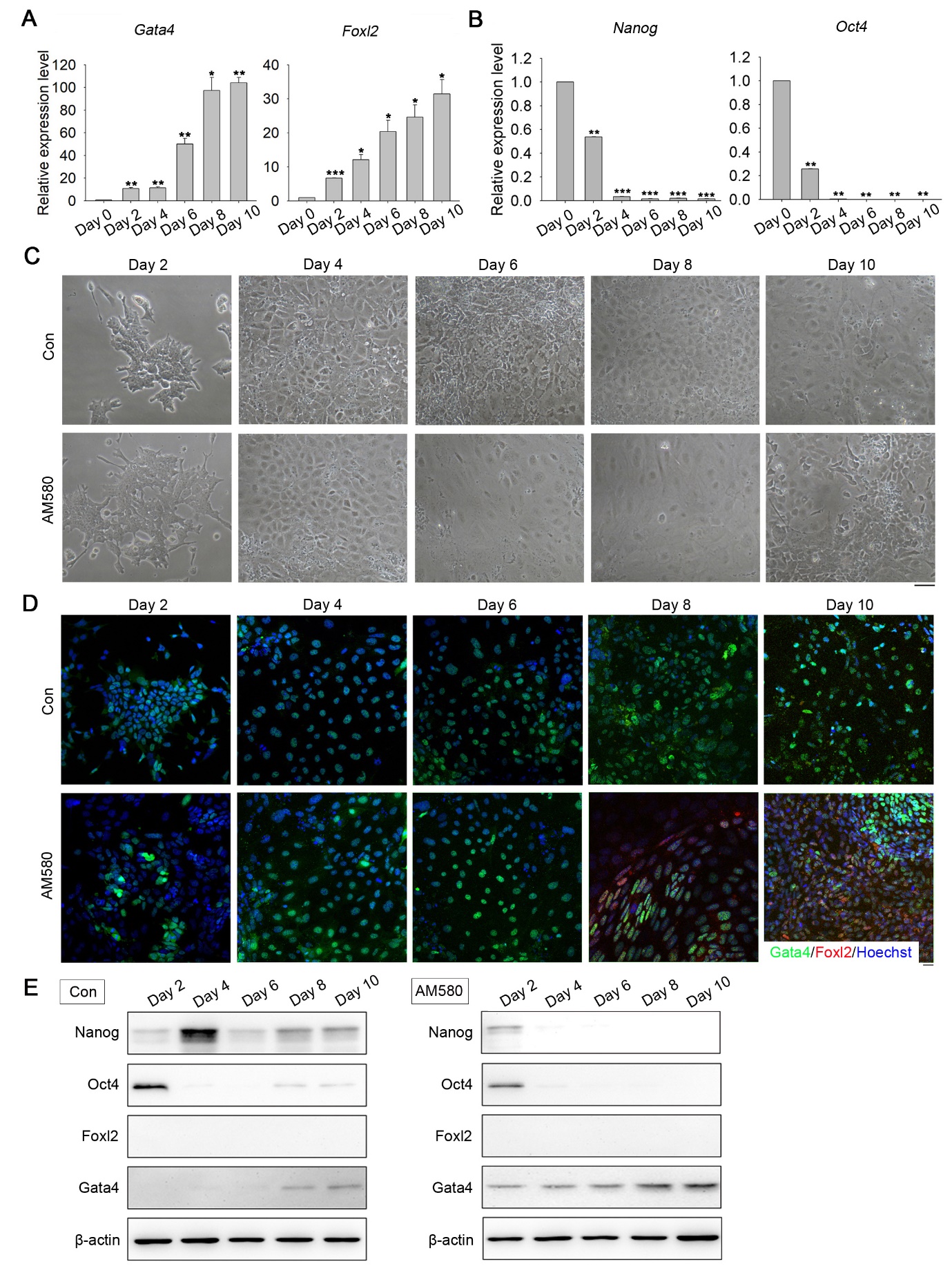


**Supplementary Figure S2. AM580 treatment induced expression of Gata4 and of Foxl2 to a lesser extent.**

(A) mRNA expression levels of *Gata4* and *Foxl2* by qPCR in GSCLCs induced by AM580 treatment. Bars = Mean± SEM (n = 3). * P <0.05, ** P <0.01, *** P <0.001.

(B) mRNA expression levels of *Nanog* and *Oct4* by qPCR in GSCLCs induced by AM580 treatment. Bars = Mean± SEM (n = 3). * P <0.05, ** P <0.01, *** P <0.001.

(C) Morphology of GSCLCs induced by Con and AM580 treatment. Scale bar = 100 μm.

(D) Immunostaining of Gata4 and Foxl2 in GSCLCs induced by AM580 treatment. Scale bar = 20 μm.

(E) Protein levels of pluripotency markers (Nanog and Oct4) and E12.5_GSCs markers (Gata4 and Foxl2) in GSCLCs were determined using western blot analysis. β-actin served as a loading control. Left: control group; right: AM580-treated group.


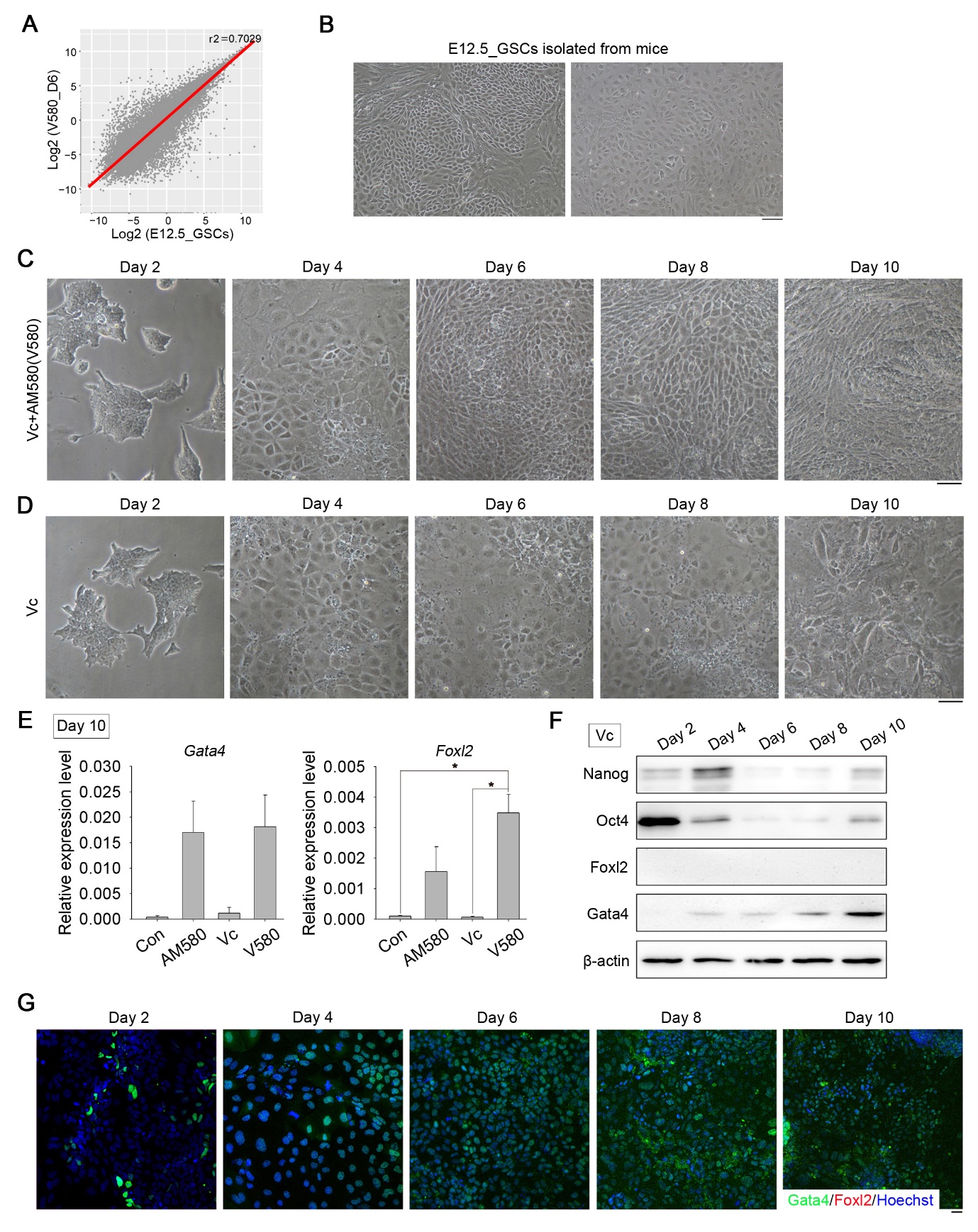


**Supplementary Figure S3. Vc alone could not induce Foxl2 expression.**

(A) Correlation analysis showed that the similarity of V580_D6 GSCLCs and E12.5_GSCs based on the overall transcriptome reached 70.29%.

(B) Morphology of E12.5_GSCs cultured in a well. Scale bar = 100 μm.

(C) Morphology of GSCLCs induced by V580 treatment. Scale bar = 100 μm.

(D) Morphology of GSCLCs induced by Vc treatment. Scale bar = 100 μm.

(E) Treatment with Vc alone did not induce *Foxl2* expression even at day 10, whereas *Gata4* was slightly upregulated. Bars = Mean± SEM (n = 3).*, P <0.05, **, P <0.01, ***, P <0.001.

(F) Protein levels of pluripotency markers (Nanog and Oct4) and E12.5_GSCs markers (Gata4 and Foxl2) in GSCLCs induced by Vc treatment, as determined using western blot analysis. β-actin served as a loading control.

(G) Immunostaining of Gata4 and Foxl2 in GSCLCs induced by Vc treatment. Scale bar = 20 μm.


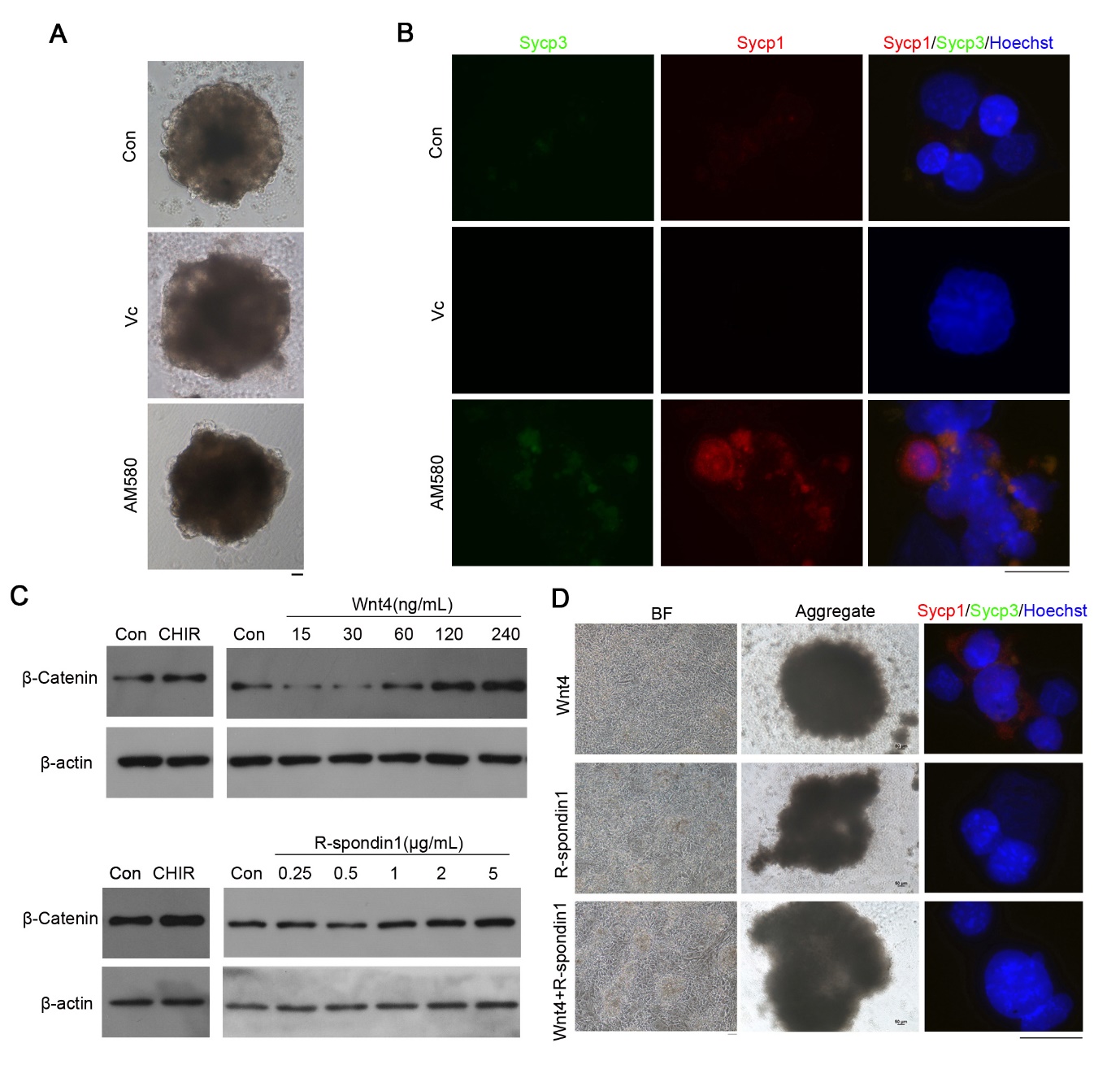
**Supplementary Figure S4. GSCLCs induced by only Vc, AM580, Wnt4, R-spondin1, or Wnt4+R-spondin1 cannot initiate meiosis.**

(A) Morphology of their aggregates induced by Vc or AM580 with E12.5_PGCs. Scale bar = 50 μm. Con: ESCs induced without molecules served as a control. Scale bar = 50 μm.

(B) No Sycp3-positive meiocytes were detected in these groups. Scale bar = 20 μm.

(C) Optimal concentrations of Wnt4 and R-spondin1 for activating the Wnt signaling pathway; 120 ng/mL Wnt4 and 5 μg/mL R-spondin1 were used.

(D) Morphology of GSCLCs induced by Wnt4 and R-spondin1 and their aggregates with PGCs. No Sycp3-positive meiocytes were detected in these groups. Left scale bar = 50 μm, middle scale bar = 50 μm, right scale bar = 20 μm.


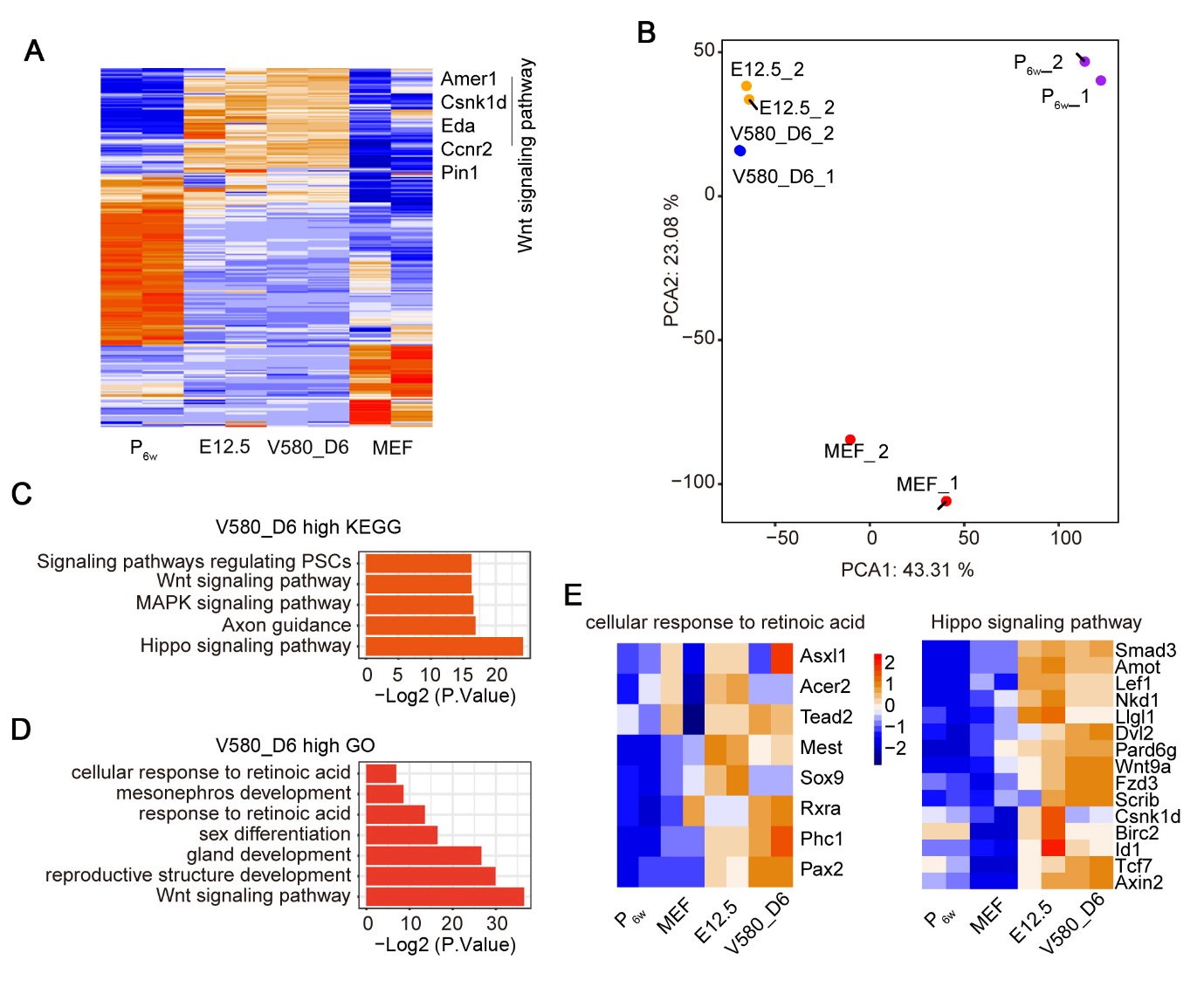


**Supplementary Figure S5. The reason why V580_D6 GSCLCs can stimulate normal meiosis progression.**

(A) Pheatmap showing DEGs in MEF, V580_D6 GSCLCs, E12.5_GSCs, and P_6w_.

(B) PCA showing similar expression between E12.5_GSCs and V580_D6 GSCLCs.

(C) KEGG analysis of pathways activated in V580_D6 GSCLCs.

(D) GO terms enriched in V580_D6 GSCLCs.

(E) Similar gene expression patterns in V580_D6 GSCLCs and E12.5_GSCs associated with the cellular response to retinoic acid and the Hippo signaling pathway.


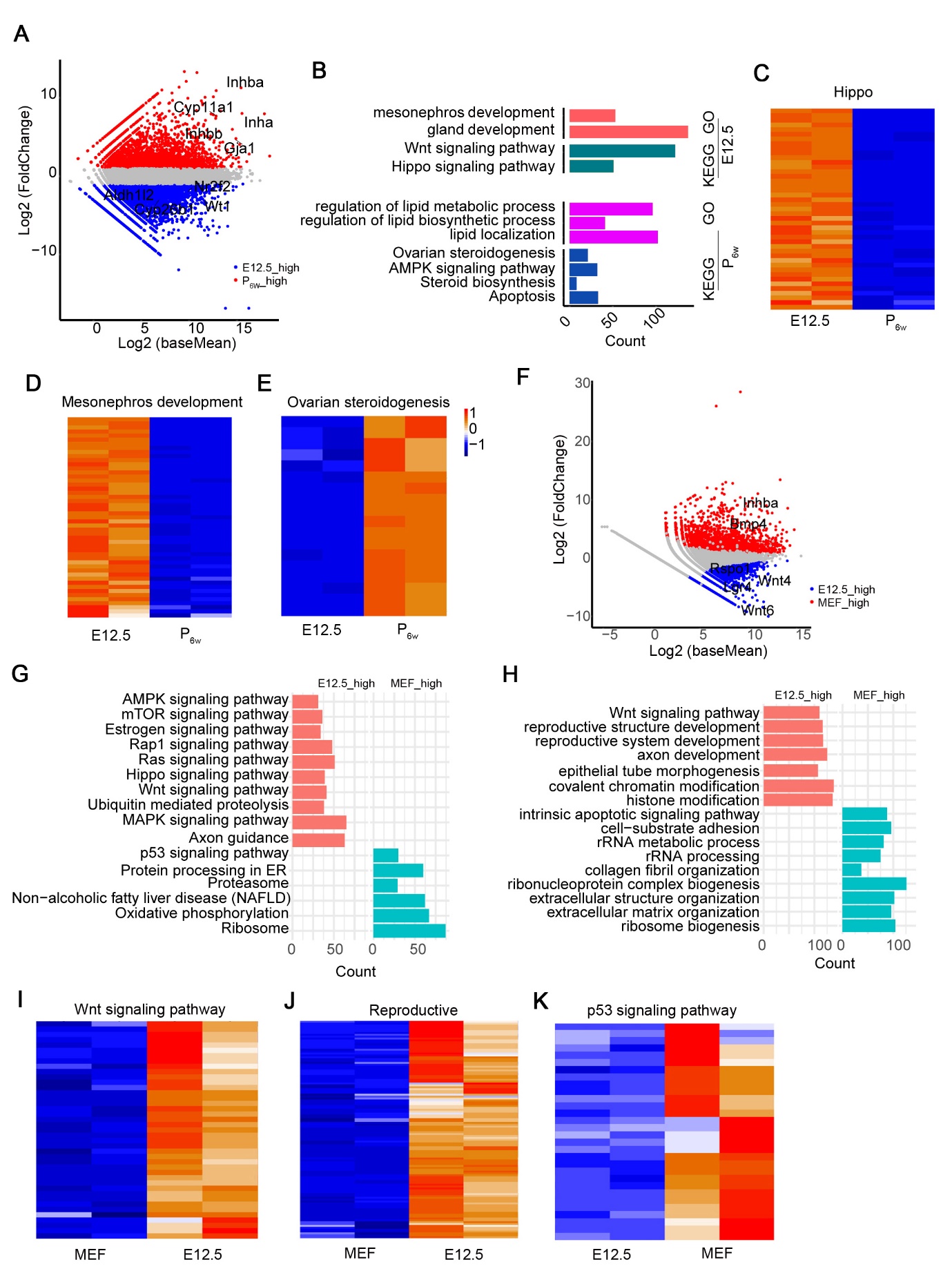


**Supplementary Figure S6. Comparison of transcriptomes between P_6w,_ MEF and E12.5_GSCs.**

(A) Scatter plot showing DEGs between E12.5 (E12.5_GSCs) and P_6w_.

(B) KEGG and GO analysis of DEGs between E12.5_GSCs and P_6w_.

(C-E) Representative GO terms in Hippo signaling, Mesonephros development, Ovarian steroidogenesis.

(F) Scatter plot showing differential gene expression between E12.5_GSCs and MEF.

(G) KEGG analysis of DEGs between E12.5_GSCs and MEF.

(H) GO analysis of DEGs between E12.5_GSCs and MEF.

(I-K) GO terms in Wnt signaling, Reproductive process and p53 signaling pathway.


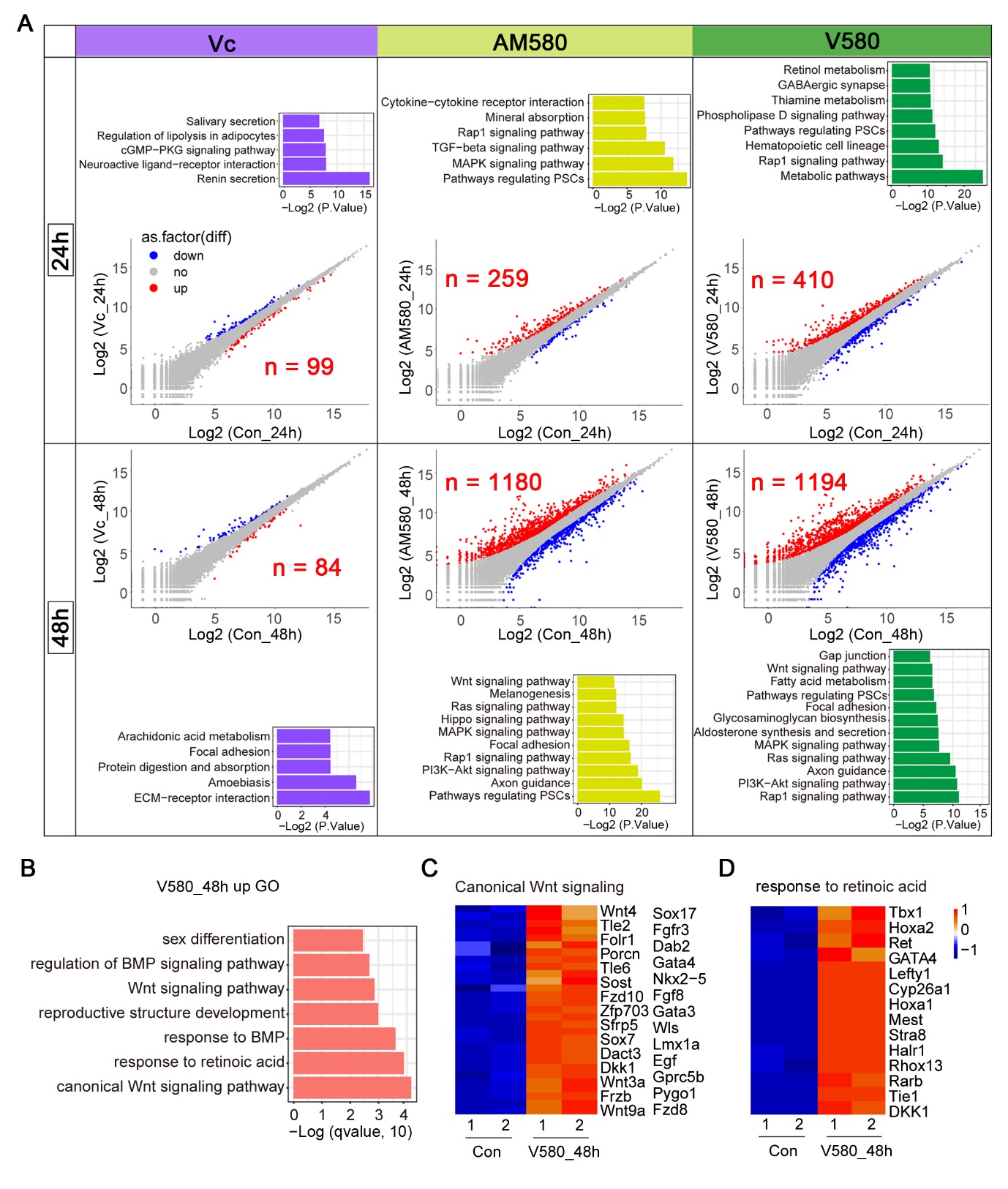


**Supplementary Figure S7: Retinoic Acid- and Wnt-related pathways were activated after V580 treatment for 48 h.**

(A) Scatterplots for global transcription and KEGG analysis of the upregulated genes. Parallel diagonal lines indicate two-fold threshold for expression differences (P < 0.05).

(B) GO analysis of upregulated genes in V580_48h.

(C) GO terms in Canonical Wnt signaling.


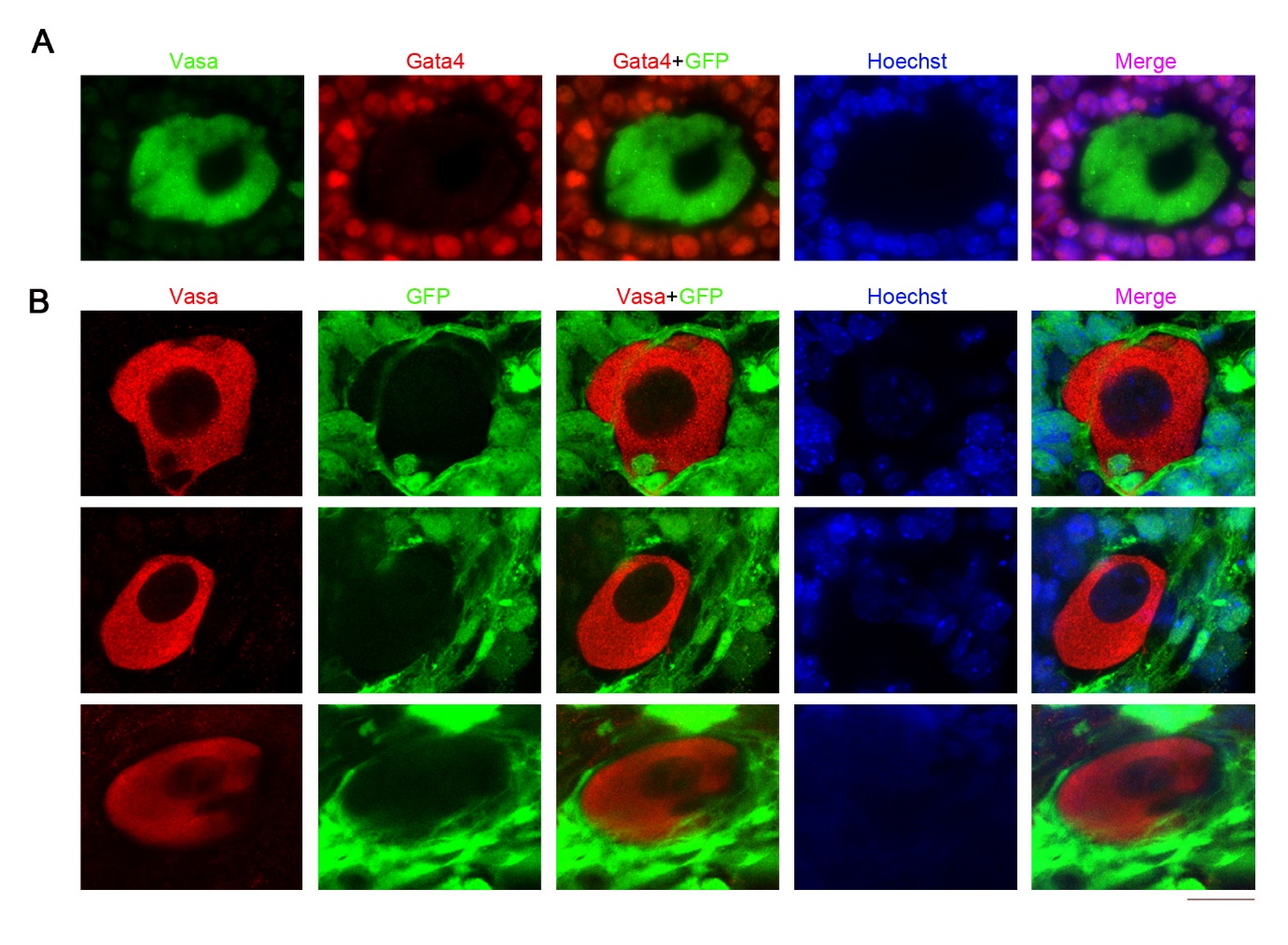
(D) GO terms in response to retinoic acid.

**Supplementary Figure S8: Vasa^+^ cells in later developmental stage in Gonad_rOvaries and CD63^+^_rOvaries.**

(A) Vasa^+^ cells in later stage in Gonad_rOvaries cultured on Transwell membranes for 21 days. D2+21: cultured in 96 well to form aggregates for 2 days, then cultured on Transwell membranes for another 21 days. Scale bar = 10 μm.

(B) Vasa^+^ cells in CD63^+^_rOvaries can develop to the later stage of ovary development. Scale bar = 10 μm. GFP: GSCLCs.

**Supplementary Table S1. Primers for qPCR analysis.**

| **Genes** | **Forward Primer** | **Reverse Primer** |
| --- | --- | --- |
| *Gapdh* | TCAACAGCAACTCCCACTCTTCCA | ACCACCCTGTTGCTGTAGCCGTAT |
| *Gata4* | GAGCTGGCCTGCGATGTCTGAGTG | AAACGGAAGCCCAAGAACCTGAAT |
| *Foxl2* | ACAACACCGGAGAAACCAGAC | CGTAGAACGGGAACTTGGCTA |
| *Nanog* | TTGCTTACAAGGGTCTGCTACT | ACTGGTAGAAGAATCAGGGCT |
| *Oct4* | TTGGGCTAGAGAAGGATGTGGTT | GGAAAAGGGACTGAGTAGAGTGTGG |
